# Pharmacological CDK4/6 inhibition reveals a p53‐dependent senescent state with restricted toxicity

**DOI:** 10.15252/embj.2021108946

**Published:** 2022-01-05

**Authors:** Boshi Wang, Marta Varela‐Eirin, Simone M Brandenburg, Alejandra Hernandez‐Segura, Thijmen van Vliet, Elisabeth M Jongbloed, Saskia M Wilting, Naoko Ohtani, Agnes Jager, Marco Demaria

**Affiliations:** ^1^ European Research Institute for the Biology of Ageing (ERIBA) University Medical Center Groningen (UMCG) Groningen The Netherlands; ^2^ Department of Medical Oncology Erasmus MC Cancer Institute Erasmus University Medical Center Rotterdam The Netherlands; ^3^ Graduate School of Medicine Osaka City University Osaka Japan

**Keywords:** CDK4/6 inhibitors, cellular senescence, chemotherapy, p53, SASP, Cancer, Cell Cycle, Signal Transduction

## Abstract

Cellular senescence is a state of stable growth arrest and a desired outcome of tumor suppressive interventions. Treatment with many anti‐cancer drugs can cause premature senescence of non‐malignant cells. These therapy‐induced senescent cells can have pro‐tumorigenic and pro‐disease functions via activation of an inflammatory secretory phenotype (SASP). Inhibitors of cyclin‐dependent kinases 4/6 (CDK4/6i) have recently proven to restrain tumor growth by activating a senescence‐like program in cancer cells. However, the physiological consequence of exposing the whole organism to pharmacological CDK4/6i remains poorly characterized. Here, we show that exposure to CDK4/6i induces non‐malignant cells to enter a premature state of senescence dependent on p53. We observe in mice and breast cancer patients that the CDK4/6i‐induced senescent program activates only a partial SASP enriched in p53 targets but lacking pro‐inflammatory and NF‐κB‐driven components. We find that CDK4/6i‐induced senescent cells do not acquire pro‐tumorigenic and detrimental properties but retain the ability to promote paracrine senescence and undergo clearance. Our results demonstrate that SASP composition is exquisitely stress‐dependent and a predictor for the biological functions of different senescence subsets.

## Graphical abstract

While senescence‐linked inflammatory phenotypes caused by classical cancer drugs can be pro‐tumorigenic, senescence induced by CDK4/6 inhibitors lacks NF‐kB‐associated secretory phenotypes and is well tolerated *in vivo*.

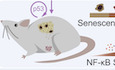

## Introduction

Unrestrained proliferation is one of the common hallmarks of cancer cells and a major target for anti‐cancer interventions (Hanahan & Weinberg, 2011). Genotoxic therapies, such as chemo‐ and radiotherapy, cause high level of DNA damage in quickly proliferating cells and often achieve tumor suppression by leading cells into senescence—a stable form of growth arrest dependent on the upregulation of cyclin‐dependent kinase (CDK) inhibitors p16 and p21 (Rodier & Campisi, 2011). A major advantage of genotoxic interventions is that they are not biased to specific molecular marks or cell types, and are effective against a broad range of tumor specimens. However, this lack of specificity and the use of systemic administration are also the basis of several short‐ and long‐term adverse reactions, which arise from inflicting unrepairable DNA damage to non‐malignant cells.

In recent years, several studies have suggested that a major mechanism leading to chemotoxicity is the premature and excessive induction of non‐malignant cells into senescence (Sun *et al*, 2012; Baar *et al*, 2017; Demaria *et al*, 2017; Murali *et al*, 2018; Yao *et al*, 2020). Genetic and pharmacological removal of non‐malignant therapy‐induced senescent (TIS) cells is sufficient to alleviate various therapy‐associated adverse reactions including fatigue, myelosuppression, cardiomyopathy, bone loss, frailty, cancer progression, and relapse (Sun *et al*, 2012; Baar *et al*, 2017; Demaria *et al*, 2017; Murali *et al*, 2018; Yao *et al*, 2020). The detrimental effects associated with TIS cells are mainly mediated by the secretion of various pro‐inflammatory cytokines and chemokine part of the complex senescence‐associated secretory phenotype (SASP; Hernandez‐Segura *et al*, 2018b). In accordance, pro‐inflammatory SASP factors are elevated in cancer patients suffering from a variety of adverse reactions to cancer therapies (Pierce *et al*, 2009; Ferrucci & Fabbri, 2018). Major regulator of pro‐inflammatory SASP genes is NF‐κB, a transcription factor mediating the response to persistent DNA damage (Rodier *et al*, 2009; Chien *et al*, 2011). However, the SASP is highly heterogeneous and regulated by additional signaling pathways, including cEBPs, NOTCH, mTOR, and SIRT1, suggesting the existence of various modules that are activated in a context‐dependent fashion (Hernandez‐Segura *et al*, 2017, 2018b). For example, cells overexpressing p16 or p53 enter a state of senescence without NF‐κB signaling and production/secretion of pro‐inflammatory and NF‐κB‐dependent SASP factors (Efeyan *et al*, 2007; Coppé *et al*, 2011; Wiley *et al*, 2018).

Another strategy to reduce tumor growth is the use of inhibitors targeting specific mechanisms that are aberrantly used by cancer cells to fuel their unrestrained proliferation. During cell cycle progression, D‐type cyclins bind to the CDK4/6, phosphorylate and inhibit the retinoblastoma (RB) tumor suppressor proteins, and derepress the activity of E2F transcription factors to allow G1‐S transition. p16, encoded by the *CDKN2A* gene, binds to CDK4/6 and inhibits cyclin–CDK complexes. As virtually all human tumor cells carry aberrations throughout the cyclin–CDK4/6–RB–p16 axis to support their hyperproliferative state, selective CDK4/6 inhibitors (CDK4/6i) have been developed in recent years (Klein *et al*, 2018). The anti‐cancer effects of CDK4/6i are mainly driven by the induction of cancer cells into senescence (Yoshida *et al*, 2016; Goel *et al*, 2017). CDK4/6i‐treated cancer cells not only stop dividing but also activate a potent tumor immunosurveillance (Goel *et al*, 2017). Palbociclib (PD033291), abemaciclib (LY2835219), and ribociclib (LEE011) are approved for the treatment of hormone‐sensitive and HER2‐negative advanced breast cancer patients, where they have been shown to improve progression‐free survival in largely metastasized breast cancer patient cohorts (Finn *et al*, 2016; Goetz *et al*, 2017). Moreover, CDK4/6i are currently investigated for treatment of different liquid and solid tumors, including as first‐line treatment for lung cancer patients (Patnaik *et al*, 2016). Systemic administration of CDK4/6i in humans has been reported to be better tolerated than genotoxic interventions (Klein *et al*, 2018). In particular, asthenia (severe fatigue) represents an important obstacle for the continuous treatment with genotoxic chemotherapy but is a rare event in patients treated with CDK4/6i (Ingham & Schwartz, 2017). Recent evidences have suggested that, similar to what observed for cancer cells, prolonged treatment with CDK4/6i can promote the premature senescence of non‐malignant cells (Guan *et al*, 2017; Hari *et al*, 2019). However, the phenotype of non‐malignant CDK4/6i‐induced senescent cells, a validation of their induction *in vivo*, and their contribution to potential therapy‐induced adverse reactions remain largely unknown. Here, using human and mouse cells, mice, and human biopsies, we aim at defining CDK4/6i‐induced senescence and its functional role.

### CDK4/6i treatment induces a state of cellular senescence dependent on p53 activity

**Figure EV1:**
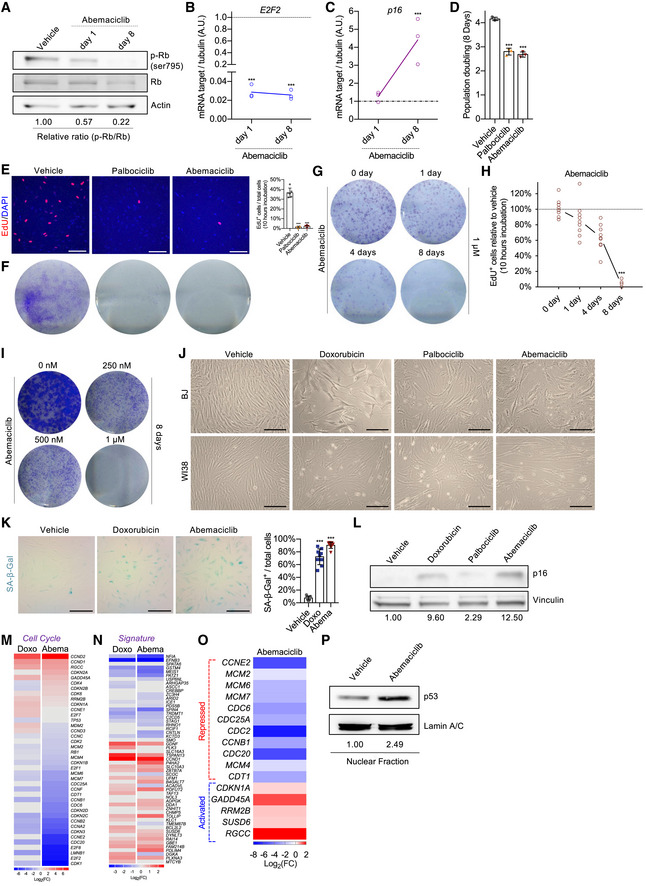
Prolonged CDK4/6i treatment induces p53‐dependent cellular senescence A–CHuman fibroblasts (BJ) were treated with vehicle (water for 8 times in 24 h) or abemaciclib (1 μM for 1 or 8 times in 24 h). Protein was isolated from vehicle or abemaciclib‐treated cells and immunoblotted for p‐Rb, Rb, and actin (A). RNA was isolated from treated cells, and mRNA levels of E2F2 gene (B) and p16 gene (C) were quantified by qPCR relative to tubulin (internal control). *n* = 3 independent experiments.D–F8‐day population doubling of WI38 cells treated with vehicle, palbociclib, or abemaciclib (both 1 μM for 8 times in 24 h; *n* = 3 independent experiments) (D). At 8 dpt, treated WI38 cells were incubated with EdU for 10 h and stained (scale bar, 150 μm; *n* = 6 samples from 3 independent experiments) (E). 3 × 103 treated WI38 cells were replated in 6‐well dish, cultured for 8 days, and stained with 0.2% crystal violet (*n* = 3 independent experiments) (F).G, HCells were treated with vehicle or abemaciclib (1 μM for 1 or 4 or 8 times in 24 h), after drug withdraw, either replated for colony formation assay (*n* = 3 independent experiments) (G) or incubated with EdU for 10 h, and stained at 8 dpt (*n* = 9 samples from 3 independent experiments) (H).ICells were treated with vehicle or abemaciclib (250 nM or 500 nM or 1 μM for 8 times in 24 h); after drug withdraw, the cells were replated for colony formation assay (*n* = 3 independent experiments).JRepresentative phase‐contrast images of BJ or WI38 cells at the end of each drug treatment (scale bar, 1 mm; *n* = 3 independent experiments).KAt 8 dpt, treated BJ cells were fixed and stained for SA‐β‐gal and quantified (scale bar, 1 mm; *n* = 3 independent experiments).LWhole‐cell lysate of treated BJ cells was used to immunoblot for p16 (*n* = 3 independent experiments).M–ORNA‐sequencing was performed with human fibroblasts (BJ) treated with vehicle (water for 8 times in 24 h) or abemaciclib (1 μM for 8 times in 24 h) (*n* = 3 independent samples, sequenced together). Heatmap of cell cycle genes (M), senescence signature (N), and p53‐repressed (red) and p53‐activated (blue) cell cycle‐related genes (O) calculated from RNA‐seq datasets of cells 8 dpt relative to the vehicle‐treated group.PCells were treated with vehicle or abemaciclib (1 μM for 8 times in 24 h); after drug withdraw, the nuclear fraction was isolated and protein extracted for Western blotting. Lamin A/C was used as the marker of nucleus and loading control (*n* = 3 independent experiments). Data information: Data are means ± SD. Two‐way ANOVA (B, C, and H). One‐way ANOVA (D, E, and K). ***P < 0.001.

**Figure 1:**
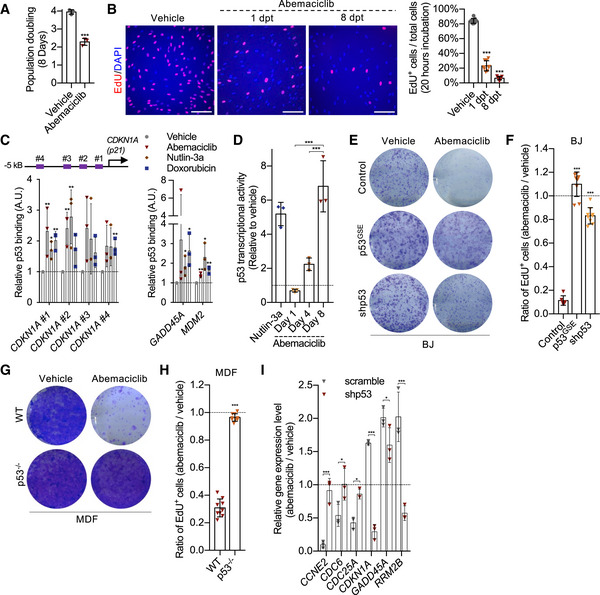
CDK4/6i treatment induces a state of cellular senescence dependent on p53 activity A, BHuman fibroblasts (BJ) were treated with vehicle (water for 8 times in 24 h) or abemaciclib (1 μM for 8 times in 24 h). The population doubling over the 8‐day treatment is plotted (A). 1 or 8 dpt, cells were incubated with EdU for 20 h and stained and quantified (*n* = 9 samples from 3 independent experiments; scale bar, 150 μm) (B).CChromatin was extracted from BJ cells treated with vehicle or abemaciclib (1 μM for 8 times in 24 h) or nutlin‐3a (10 μM for 8 times in 24 h) or doxorubicin (250 nM for 24 h), and ChIP assays using an antibody against p53 were performed. qRT–PCR was performed using primers amplifying promoter regions of *CDKN1A*, *GADD45A*, and *MDM2* genes containing p53 binding sites. Values indicate fold enrichment relative to the vehicle group (*n* = 3 independent experiments).DBJ cells transduced with a p53 reporter were treated with nutlin‐3a (10 μM, positive control) or abemaciclib (1 μM for 1 or 4 or 8 times in 24 h), and luciferase activity was measured after treatments at the indicated time points (*n* = 3 independent experiments).E3 × 10^3^ BJ cells of the indicated genotypes were replated after vehicle or abemaciclib treatment (1 μM) and stained with 0.2% crystal violet 8 dpt (*n* = 3 independent experiments).F8 dpt, treated BJ cells were incubated with EdU for 20 h and stained (*n* = 9 samples from 3 independent experiments).G3 × 10^3^ mouse dermal fibroblasts (MDFs) of the indicated genotypes were replated after vehicle or abemaciclib treatment (4 μM for 8 times in 24 h) and stained with 0.2% crystal violet 8 dpt (*n* = 3 independent experiments).H8 dpt, treated MDFs were incubated with EdU for 20 h and stained (*n* = 9 samples from 3 independent experiments).IRNA was isolated from vehicle‐ or abemaciclib (1 μM)‐treated scramble/shp53 BJ cells, and mRNA for the indicated genes was quantified by qRT–PCR relative to tubulin (*n* = 3 independent experiments). Data information: Data are means ± SD. Unpaired Student’s *t*‐test (A and H). One‐way ANOVA (B, D, and F). Two‐way ANOVA (C and I). *P < 0.05, **P < 0.01, and ***P < 0.001. dpt, days post‐treatment.

**Figure EV2:**
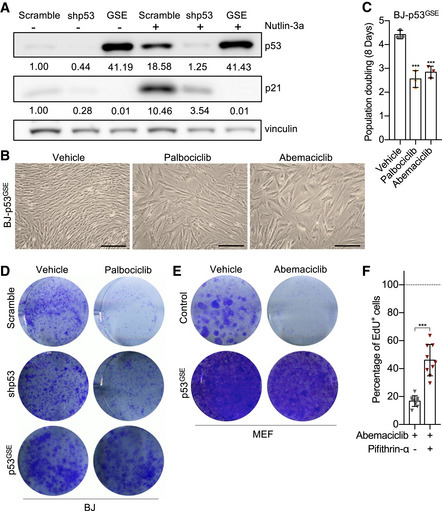
CDK4/6i induces cellular senescence dependent on p53 Immunoblot of p53 on cells of indicated genotypes and treatments.Representative images of p53^GSE^ BJ cells at the end of the 8‐day indicated treatments (scale bar, 1 mm; *n* = 3 independent experiments).8‐day population doubling of treated p53^GSE^ BJ cells (*n* = 3 independent experiments).3 × 10^3^ vehicle or palbociclib‐treated (1 μM for 8 times in 24 h) scramble/shp53/ p53GSE BJ cells were replated in 6‐well dishes, cultured for 8 days, and stained with 0.2% crystal violet (*n* = 3 independent experiments).3 × 10^3^ vehicle‐ or abemaciclib‐treated (4 μM for 8 times in 24 h) WT/p53GSE MEFs were replated in 6‐well dishes, cultured for 8 days and stained with 0.2% crystal violet (*n* = 3 independent experiments).BJ cells were treated with abemaciclib ± pifithrin‐α and EdU staining performed at 8 dpt (*n* = 9 samples from 3 independent experiments). Data information: Data are means ± SD. One‐way ANOVA (C). Unpaired two‐tailed *t*‐test (F). ***P < 0.001.

In accordance with previous studies (Yang *et al*, 2017), prolonged treatment of human primary fibroblasts (BJ) with the CDK4/6i abemaciclib was associated with a progressive loss of RB phosphorylation (Fig EV1A), downregulation of *E2F2* (Fig EV1B), and increased *p16* gene expression (Fig EV1C). Reduced population doubling (Fig 1A) and EdU incorporation (Fig 1B) at the end of treatment demonstrated the efficacy of abemaciclib to induce cell cycle arrest. A similar proliferative arrest was observed in human primary fibroblasts WI38 treated with abemaciclib or another CDK4/6i, palbociclib (Fig EV1D). Importantly, drug withdrawal was not sufficient to restore proliferation of CDK4/6i‐treated cells (Figs 1B and EV1E and F), particularly after an 8‐day treatment at 1 μM (Fig EV1G–I). CDK4/6i‐treated cells assumed a flattened and enlarged morphology (Fig EV1J), activated the senescence‐associated‐β‐galactosidase (SA‐β‐gal) (Fig EV1K), and maintained high level of the endogenous CDK4/6i p16 (Fig EV1L), all features that can also be observed in cells treated with other anti‐cancer and senescence‐inducing agents such as the genotoxic drug doxorubicin (Demaria *et al*, 2017). RNA‐sequencing analysis of cells 8 days post‐treatment revealed that downregulation of cell cycle genes (Fig EV1M), upregulation of lysosomal‐associated pathways (Table EV1), and activation of a “core” transcriptional signature of senescence previously identified in our laboratory (Hernandez‐Segura *et al*, 2017) (Fig EV1N) were observed in both abemaciclib‐ and doxorubicin‐treated cells. Interestingly, RNA‐sequencing data suggested that abemaciclib‐treated BJ cells differentially expressed several p53 transcriptional targets related to cell cycle (Spurgers *et al*, 2006; Fischer, 2017; Fig EV1O). In accordance, we observed an increased nuclear localization of p53 in abemaciclib‐treated BJ cells (Fig EV1P), while ChIP analyses revealed that abemaciclib enhanced p53 binding to the promoter regions of its target genes *CDKN1A (p21)*, *MDM2*, and *GADD45A*, which was similar to what observed in cells treated with doxorubicin or nutlin‐3a (Fig 1C). In addition, BJ cells with stable expression of a luciferase reporter construct (p53LUC) confirmed gradual activation of p53 transcriptional activity upon exposure to abemaciclib, similar to what was observed in cells treated with the Mdm2 inhibitor nutlin‐3a (Fig 1D). To evaluate whether p53 was essential to orchestrate the irreversible cell cycle arrest in response to CDK4/6i, we analyzed various models with impaired p53 expression or activity. BJ cells transduced with a genetic suppressor element (GSE) against p53 (Mittelman & Gudkov, 1999) or with shRNA lentiviral particles (Fig EV2A) assumed enlarged morphology (Fig EV2B) and activated a temporary arrest (Fig EV2C) during abemaciclib or palbociclib treatment, comparable to what observed in wild‐type cells. However, in the absence of p53, cells were able to restore proliferation after drug removal, as measured by colony formation assays (Figs 1E and EV2D) and EdU incorporation (Fig 1F). Similarly, mouse dermal fibroblasts (MDFs) derived from p53 knockout mice (Fig 1 G and H) and wild‐type mouse embryonic fibroblasts (MEFs) with inactive p53 (Fig EV2E) also failed to enter irreversible growth arrest upon exposure to abemaciclib. The bypass of irreversible growth arrest in p53‐deficient BJ cells was associated with a failure in modulating the expression of several p53 transcriptional targets related to cell cycle upon treatment with abemaciclib (Fig 1I). Finally, to further evaluate the dependence of CDK4/6‐induced irreversible growth arrest on p53 transcriptional activity, we exposed BJ cells to pifithrin‐α (PFT‐α), a reversible inhibitor of p53‐dependent transcription (Sohn *et al*, 2009). The concomitant treatment abemaciclib/PFT‐α allowed a substantial number of BJ cells to bypass the irreversible growth arrest (Fig EV2F). Taken together, these data suggest that CDK4/6i induce non‐malignant cells to a state of a senescence‐associated permanent growth arrest dependent on p53 transcriptional activity.

### CDK4/6i promotes premature senescent cells *in vivo* without toxicity

**Figure EV3:**
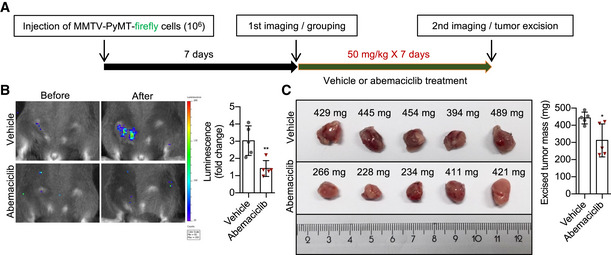
Senescence‐inducing dose of abemaciclib inhibits tumor growth Scheme of abemaciclib treatments for MMTV‐PyMT‐firefly breast cancer mouse model *in vivo*.Female p16‐3MR mice bearing MMTV‐PyMT‐firefly tumors in mammary fat pad were treated with vehicle (PBS, 7 consecutive days) or abemaciclib (50 mg/kg in PBS, 7 consecutive days). The mice were injected with D‐Luciferin, and bioluminescence was visualized/quantified by the IVIS spectrum *in vivo* imaging system before and after abemaciclib treatments, as shown by representative images and quantification (*n* = 5 mice/group).Excised tumors and quantification of tumor weights (*n* = 5 mice/group). Data information: Data are means ± SD. Unpaired two‐tailed *t*‐test (B and C). *P < 0.05 and **P < 0.01.

**Figure 2:**
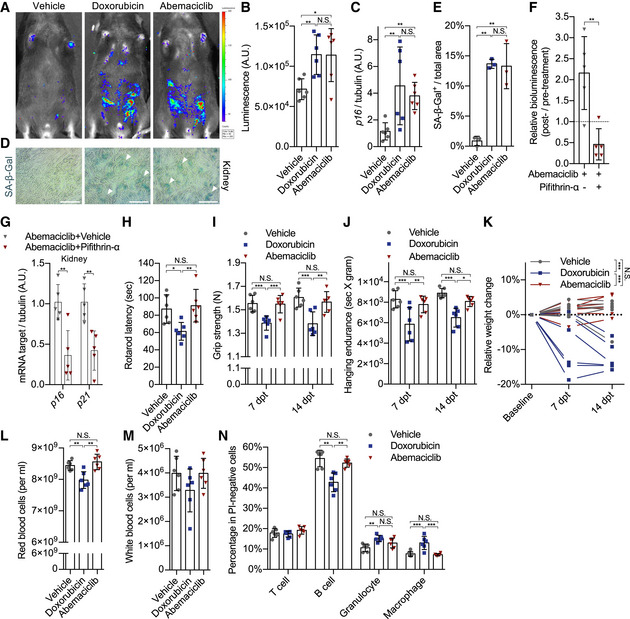
CDK4/6i promotes premature senescent cells *in vivo* without toxicity A–Ep16‐3MR mice were treated with vehicle (PBS, 7 consecutive days), doxorubicin (5 mg/kg, 3 consecutive days), or abemaciclib (50 mg/kg, 7 consecutive days). *n* = 6 mice/group. 14 dpt, bioluminescence was visualized and quantified by the IVIS spectrum *in vivo* imaging system, as shown by representative bioluminescence images (A) and quantification (B). RNA was isolated from kidneys, and mRNA encoding p16 was quantified by qRT–PCR (C). Representative images (D) to visualize SA‐β‐gal activities in vehicle‐, doxorubicin‐, or abemaciclib‐treated mouse kidney sections at 15 dpt (arrows indicated positive area; scale bar, 1 mm; *n* = 3 mice/group) and quantified (E).Fp16‐3MR mice were treated with abemaciclib (50 mg/kg, 7 consecutive days) ± pifithrin‐α (2 mg/kg, 7 consecutive days). Values indicate the ratio of post‐treatment bioluminescence to pretreatment (*n* = 5 mice/group).GRNA was isolated from abemaciclib ± pifithrin‐α treated kidneys, and mRNA encoding *p16* and *p21* genes was quantified by qRT–PCR and normalized on tubulin (*n* = 5 mice/group).H–NFor the doxorubicin‐ or abemaciclib‐treated mice (*n* = 6 mice/group), physical performance was measured by rotarod assay at 15 dpt (H), and grip strength meter at 7 dpt and 14 dpt (I), and hanging tests were performed at 7 dpt and 14 dpt and normalized to weights (J). (K) Relative weight changes were calculated at 7 dpt and 14 dpt. Red blood cells (L) and white blood cells (M) were counted at 15 dpt. Percentage of T cells, B cells, granulocytes, and macrophages were determined by flow cytometry analysis (N). Data information: Data are means ± SD. One‐way ANOVA (B, C, E, H, L, and M). Unpaired Student’s *t*‐test. (F). Two‐way ANOVA (G, I, J, K, and N). *P < 0.05, **P < 0.01, and ***P < 0.001, N.S. = not significant. dpt, days post‐treatment.

To determine whether induction of normal cells into senescence upon treatment with CDK4/6i happens *in vivo*, we exposed mice to a clinically relevant and tumor‐suppressive dose of abemaciclib. At first, the anti‐neoplastic effects of this dose (50 mg/kg/day of abemaciclib for 7 consecutive days) were validated in our laboratory using a model of breast cancer (Fig EV3A–C). Then, we treated cancer‐free p16‐3MR mice, which harbor a Renilla luciferase (RL) reporter gene driven by the p16 promoter (Demaria *et al*, 2014), with abemaciclib or doxorubicin. Abemaciclib‐ and doxorubicin‐treated mice showed a similar enhanced bioluminescent signal (Fig 2A and B), and comparable induction of p16 (Fig 2C) and SA‐β‐gal in kidneys (Fig 2D and E). To evaluate whether induction to senescence by CDK4/6i treatment depends on p53 also *in vivo*, we exposed p16‐3MR mice to a co‐treatment abemaciclib/PFT‐α. The presence of the p53 transcriptional inhibitor PFT‐α prevented abemaciclib‐mediated induction of whole‐body luminescence (Fig 2F) and of p16 and p21 expression in kidney (Fig 2G), suggesting senescence bypass. Therapy‐induced senescence is an important promoter of several adverse reactions to treatment (Wang *et al*, 2020) independent from tumorigenesis. Among those, evidences from preclinical and clinical contexts have highlighted the correlation between levels of senescence and severe fatigue (Wang *et al*, 2020). To evaluate whether such detrimental effect is also exerted by CDK4/6i‐induced senescent cells, we compared the physical activity of cancer‐free mice exposed to abemaciclib or doxorubicin (Fig 2A–E). As previously shown (Demaria *et al*, 2017), treatment with doxorubicin severely affected strength and endurance, as measured by rotarod assay (Fig 2H), grip strength (Fig 2I), and hanging time (Fig 2J) at both 7 and 14 days after treatment. In contrast, mice treated with abemaciclib did not show any apparent reduction of physical performance in comparison with vehicle‐treated cohorts (Fig 2 H–J). Weight and blood counts of abemaciclib‐treated mice remained similar to control mice, while doxorubicin‐treated animals showed substantial weight loss (Fig 2K), reduced number of red blood cells (Fig 2L), a slight decrease in the total number of leukocytes (Fig 2M), and impaired proportion of B cells, granulocytes, and macrophages (Fig 2N). Together, these data suggest that treatment with CDK4/6i induces a well‐tolerated state of senescence *in vivo*.

### CDK4/6i‐induced senescence lacks NF‐κB activity and NF‐κB‐driven SASP components

**Figure EV4:**
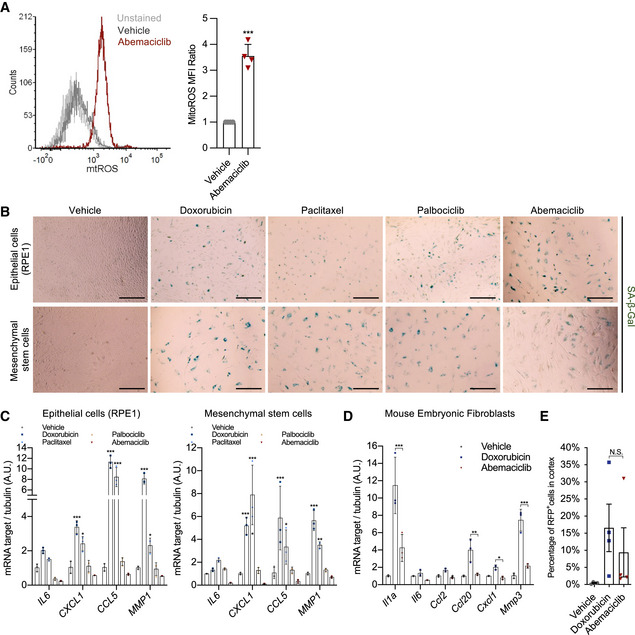
CDK4/6i induces cellular senescence without pro‐inflammatory SASP BJ cells were treated with vehicle or abemaciclib and stained for mitochondria ROS. Relative values of ROS level were plotted (*n* = 4 independent experiments).Human hTERT‐RPE1 and lung mesenchymal stem cells were treated with vehicle (DMSO for 8 times in 24 h) or doxorubicin (250 nM for 24 h) or paclitaxel (50 nM for 24 h) or palbociclib (1 μM for 8 times in 24 h) or abemaciclib (1 μM for 8 times in 24 h). At 8 dpt, treated cells were fixed and stained for SA‐β‐gal (scale bar, 1 mm; *n* = 3 independent experiments).At 8 dpt, RNA was isolated from treated cells and indicated NF‐κB‐associated SASP (NASP) genes were quantified by qRT–PCR relative to tubulin (*n* = 3 independent experiments).qRT–PCR of indicated genes was performed using mouse embryonic fibroblast (MEFs) 8 days after the indicated treatments (*n* = 3 independent experiments).RFP^+^ cells were sorted from renal cortex of doxorubicin‐ or abemaciclib‐treated p16‐3MR mice. The percentage of RFP^+^ cells in total cells was plotted (*n* = 4 mice/group). Data information: Data are means ± SD. Two‐way ANOVA (C and D). One‐way ANOVA (E). *P < 0.05, **P < 0.01, and ***P < 0.001, N.S. = not significant.

**Figure 3:**
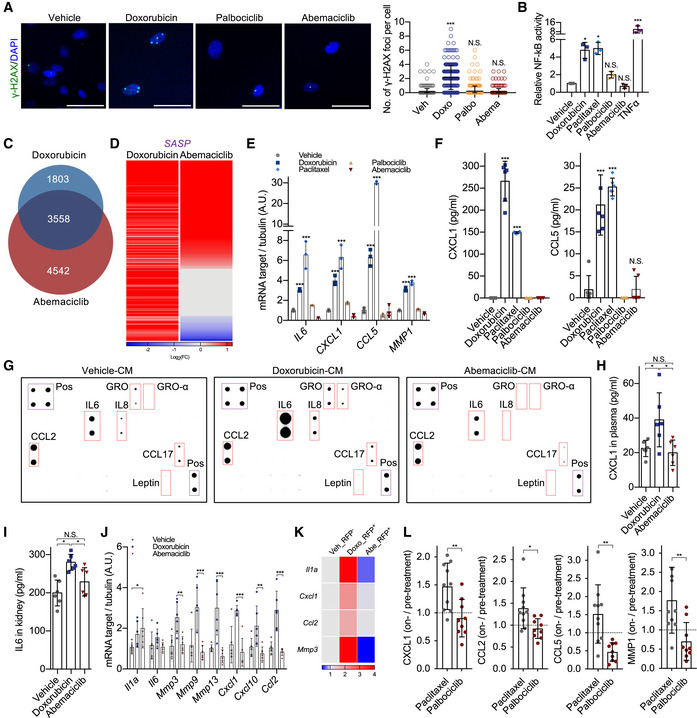
CDK4/6i‐induced senescence lacks NF‐κB activity and NF‐κB‐driven SASP components A8 dpt, cells were stained for γ‐H2AX (scale bar, 60 μm) and quantified (vehicle = 288 cells, doxorubicin = 280 cells, palbociclib = 276 cells, abemaciclib = 266 cells from 3 independent experiments).BBJ cells transduced with a NF‐κB reporter were treated with vehicle, doxorubicin, paclitaxel (50 nM for 24 h), palbociclib (1 μM for 8 times in 24 h), or abemaciclib, and luciferase activity was measured 8 dpt. TNF‐α treatment was used as positive control (*n* = 3 independent experiments).C, DVenn plot of RNA‐sequencing datasets of treated cells at 8 dpt (*n* = 3 independent samples, sequenced together) (C). Heatmap of the SASP genes (D) (Table EV2) for doxorubicin‐ or abemaciclib‐treated groups (8 dpt) relative to the vehicle‐treated group from the RNA‐sequencing datasets (*P* < 0.01 was regarded as significant).E8 dpt, qRT–PCR of indicated NF‐κB‐associated SASP genes was performed using indicated treated BJ cells 8 dpt (*n* = 3 independent experiments).FSerum‐free conditioned media (CM) were collected from vehicle‐, doxorubicin‐, paclitaxel‐, palbociclib‐, or abemaciclib‐treated BJ cells 8 dpt, and expression levels of CXCL1 and CCL5 were measured by ELISA (*n* = 6 independent experiments).GProteins in the serum‐free conditioned media (CM) collected from doxorubicin‐ or abemaciclib‐treated BJ fibroblasts (8 dpt) were measured by cytokine array. Purple boxes indicated internal positive controls from each membrane; red boxes highlighted the most typical SASP factors.H–Kp16‐3MR mice were treated with vehicle (PBS, 7 consecutive days), doxorubicin (5 mg/kg, 3 consecutive days), or abemaciclib (50 mg/kg, 7 consecutive days). 15 dpt, plasma was collected and expression levels of CXCL1 were measured by ELISA (H); protein lysate was obtained from drug‐treated kidneys to quantify IL‐6 by ELISA (I); and RNA was isolated from kidneys, and mRNA encoding indicated NF‐κB‐associated genes (J) were quantified by qRT–PCR. *n* = 6 mice/group. From a subset of the mice, RFP^+^ cells were sorted from the renal cortex using FACS and mRNA encoding indicated NF‐κB‐associated pro‐inflammatory genes were quantified by qRT–PCR. The values of fold change over vehicle were plotted in the heatmap. *n* = 4 mice/group (K).LCell‐free plasma was derived from breast cancer patients treated with paclitaxel (*n* = 10) or palbociclib (*n* = 9) and the expression levels of CXCL1, CCL2, CCL5, and MMP1 were quantified by ELISA. The values represent the ratio between on‐treatment and pretreatment levels for each patient. Data information: Data are means ± SD. One‐way ANOVA (A, B, F, H and I). Two‐way ANOVA (E and J). Unpaired Student’s *t*‐test (L). *P < 0.05, **P < 0.01, and ***P < 0.001, N.S. = not significant. Doxo, doxorubicin. Abe, abemaciclib. dpt, days post‐treatment.

Abemaciclib‐treated cells showed increased levels of mitochondrial ROS, which could explain higher p53 activity (Fig EV4A). However, the level of DNA damage response (DDR) signaling was absent from CDK4/6i‐induced senescence (Fig 3A). In senescent cells, DDR signaling is a major activator of NF‐κB, which in normal cells is dispensable for the cell cycle arrest but acts as a strong inducer of pro‐inflammatory SASP factors (Coppé *et al*, 2008; Chien *et al*, 2011). Interestingly, cells induced to senescence by palbociclib or abemaciclib did not enhance NF‐κB activity, as quantified via a luminescent reporter system, whereas a strong upregulation was observed in cells exposed to either doxorubicin or paclitaxel (Fig 3B). This was in accordance with RNA‐sequencing data, which indicated that a vast majority of NF‐κB‐associated SASP genes were up‐regulated in genotoxic stress‐induced senescent cells (doxorubicin), while these genes were not differentially regulated or even downregulated during CDK4/6i‐induced senescence (Fig 3C and D and Table EV2). qPCR (Fig 3E), ELISAs (Fig 3F) and cytokine array (Fig 3G) validated and confirmed that in primary human fibroblasts, only genotoxic drugs, but not CDK4/6i, were able to promote the production and secretion of factor part of the NF‐κB‐associated secretory phenotype (from now called NASP) such as IL‐6, CXCL1, CCL5, and MMP1. This difference was also observed in cell types other than fibroblasts, including the epithelial cells RPE1 and lung mesenchymal stem cells (MSCs) (Fig EV4B and C), and also in primary mouse fibroblasts (Fig EV4D). At next, we compared the NASP levels in mice treated with abemaciclib or doxorubicin. Doxorubicin treatment increased the concentration of CXCL1/KC in mouse plasma (Fig 3H) and promoted expression of IL‐6 (Fig 3I) and various other NASP factors in kidneys (Fig 3J), while abemaciclib had no effects. To further evaluate the NASP *in vivo*, we repeated the experiment using p16‐3MR mice. 7–14 days after doxorubicin or abemaciclib treatment, we purified RFP^+^ (p16^+^) cells from the renal cortex. As expected, the number of RFP^+^ cells was similarly increased in doxorubicin‐ and abemaciclib‐treated tissues (Fig EV4E). However, in line with what previously observed, upregulation of NASP factors was only observed in doxorubicin‐treated animals (Fig 3K). Finally, to validate these findings in a clinically relevant system, we measured the NASP in breast cancer patients prior to and after treatment with either chemotherapy or CDK4/6i. Strikingly, the plasma level of the NASP factors CXCL1, CCL2, CCL5 and MMP1 increased after treatment with paclitaxel, but no induction was observed for patients under palbociclib treatment (Fig 3L). Altogether, these data demonstrate that treatment with CDK4/6i causes non‐malignant cells to enter a state of senescence without NF‐κB signaling and NASP, the NF‐κB‐driven secretory program.

### p53‐associated SASP factors are induced during CDK4/6i‐induced senescence

**Figure 4:**
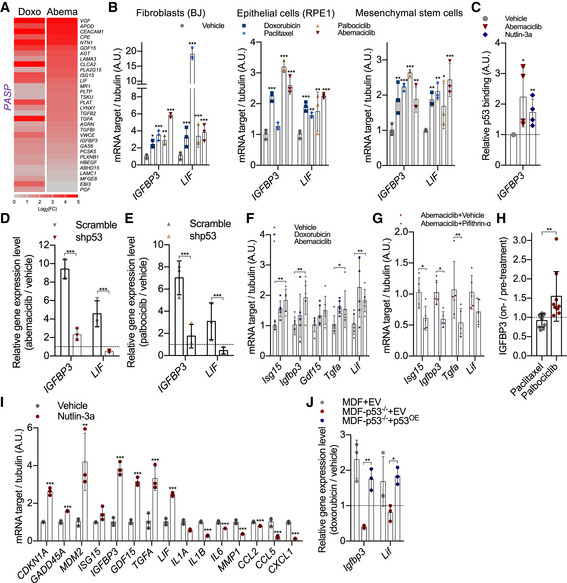
p53‐associated SASP factors are induced during CDK4/6i‐induced senescence AHuman fibroblasts (BJ) were treated with vehicle (water for 8 times in 24 h), abemaciclib (1 μM for 8 times in 24 h), or doxorubicin (250 nM for 24 h). RNA‐sequencing was performed with treated cells at 8 dpt (*n* = 3 independent samples, sequenced together) and heatmap of the p53‐associated SASP for doxorubicin‐ or abemaciclib‐treated groups (8 dpt) relative to vehicle‐treated group from the RNA‐sequencing datasets (*P* < 0.01 was regarded as significant).BHuman BJ fibroblasts, hTERT‐RPE1 epithelial cells, and lung mesenchymal stem cells were treated with vehicle (DMSO for 8 times in 24 h) or doxorubicin (250 nM for 24 h) or paclitaxel (50 nM for 24 h) or palbociclib (1 μM for 8 times in 24 h) or abemaciclib (1 μM for 8 times in 24 h). At 8 dpt, RNA was isolated and mRNA for the indicated p53‐associated SASP genes was quantified by qRT–PCR relative to tubulin (*n* = 3 independent experiments).CChromatin was extracted from BJ fibroblasts treated with vehicle or abemaciclib or nutlin‐3a, and ChIP assays using an antibody against p53 were performed. qRT–PCR was performed using primers amplifying the promoter region of *IGFBP3* containing p53 binding sites. Values indicate fold enrichment relative to the vehicle group (*n* = 4 independent experiments).D, ERNA was isolated from vehicle‐, abemaciclib (1 μM)‐ (D), or palbociclib (1 μM) (E)‐treated scramble/shp53 BJ cells and quantified by qRT–PCR for *IGFBP3* or *LIF* genes (*n* = 3 independent experiments).FRNA was isolated from kidneys treated with doxorubicin or abemaciclib, and mRNA encoding indicated p53‐associated SASP genes were quantified by qRT–PCR (*n* = 6 mice/group).GRNA was isolated from kidneys of p16‐3MR mice treated with abemaciclib (50 mg/kg, 7 consecutive days) ± pifithrin‐α (2 mg/kg, 7 consecutive days), and mRNA encoding p53‐associated SASP genes were quantified by qRT–PCR and normalized on tubulin (*n* = 5 mice/group).HCell‐free plasma was derived from breast cancer patients treated with paclitaxel (*n* = 10) or palbociclib (*n* = 9), and the expression levels of IGFBP3 were quantified by ELISA. The values represent the ratio between on‐treatment and pretreatment levels for each patient.IRNA was isolated from vehicle‐ or nutlin‐3a (1 μM for 8 times in 24 h)‐treated BJ cells at 8 dpt and quantified by qRT–PCR for p53 target cell cycle genes, SASP genes (*n* = 3 independent experiments).JMouse dermal fibroblasts (MDF) or MDF‐p53^−/−^ fibroblasts were transfected with empty vector (EV) or p53‐overexpressing vector (p53^OE^); then, the cells were treated with vehicle (water) or doxorubicin (250 nM for 24 h) and qRT–PCR was performed for *Igfbp3* and *Lif* genes (*n* = 3 independent experiments). Data information: Data are means ± SD. Two‐way ANOVA (B, D, E, F, G, I, and J). One‐way ANOVA (C). Unpaired Student’s *t*‐test (H). *P < 0.05, **P < 0.01, and ***P < 0.001. EV, empty vector, dpt, days post‐treatment.

RNA‐sequencing data suggested that although abemaciclib‐treated cells did not induce expression of pro‐inflammatory factors, other previously described SASP factors were similarly upregulated in both CDK4/6i‐induced and genotoxic stress‐induced senescent cells. Interestingly, among these SASP factors, several were predicted to be p53 target (Fischer, 2017; Fig 4A). Using the SASP factors *IGFBP3* and *LIF* as representative p53 targets, we measured their levels by qPCR in BJ, RPE1, and MSC cells exposed to senescence‐inducing doses of the CDK4/6i palbociclib and abemaciclib or the genotoxic drugs paclitaxel and doxorubicin, and validated the observations made from the RNA‐seq data (Fig 4B). ChIP analyses revealed that abemaciclib enhanced p53 binding to the promoter region of *IGFBP3* (Buckbinder *et al*, 1995), similar to what observed upon exposure to nutlin‐3a (Fig 4C). Third, the treatment with abemaciclib (Fig 4D) or palbociclib (Fig 4E) of BJ cells transduced with shRNA lentiviral particles against p53 failed to induce expression of *LIF* and *IGFBP3*. The p53‐associated SASP factors *Isg15*, *Igfbp3*, *Gdf15*, *Tgfa*, and *Lif* were equally upregulated in the kidneys of mice exposed to doxorubicin or abemaciclib (Fig 4F). Moreover, the increased level of these factors could be compromised by the co‐treatment with pifithrin‐α (PFT‐α) (Fig 4G). Interestingly, increased levels of IGFBP3 could be detected in the plasma of breast cancer patients after treatment with palbociclib (Fig 4H). While p53 is thought to interfere with the production of pro‐inflammatory SASP factors (Coppé *et al*, 2008), less is known about the existence of a p53‐associated secretory phenotype (from now called PASP). To further explore this possibility, we used two different senescence systems. First, we measured the levels of potential PASP factors in cells treated with nutlin‐3a, a treatment that leads primary cells to engage a p53‐dependent but NF‐κB‐free senescence program (Wiley *et al*, 2018). Continuous treatment with Nutlin‐3a induced BJ cells into senescence, as measured by high level of *p21*, *GADD45* and *MDM2* and an irreversible growth arrest (Fig 4I). Importantly, Nutlin‐3a‐induced senescence was associated with high expression of the PASP factors *ISG15*, *IGFBP3*, *GDF15*, *TGFA*, and *LIF*, while no induction of NASP factors was observed (Fig 4I). Second, we measured the level of the PASP factors *LIF* and *IGFBP3* in p53 WT or KO mouse dermal fibroblasts (MDFs) exposed to senescence‐inducing doses of genotoxic agents. Doxorubicin treatment failed to promote the transcription of *LIF* and *IGFBP3* in p53^‐/‐^ MDFs, but the induction was rescued in p53^‐/‐^ MDFs transduced with an expression vector carrying WT p53 (Fig 4J). Together, these data show the existence of a SASP module positively regulated by the transcriptional activity of p53 defined the PASP, which is equally promoted during genotoxic stress‐induced and CDK4/6i‐induced senescence.

### CDK4/6i‐induced senescence lacks pro‐tumorigenic properties but retain ability to promote paracrine senescence and undergo clearance

**Figure 5:**
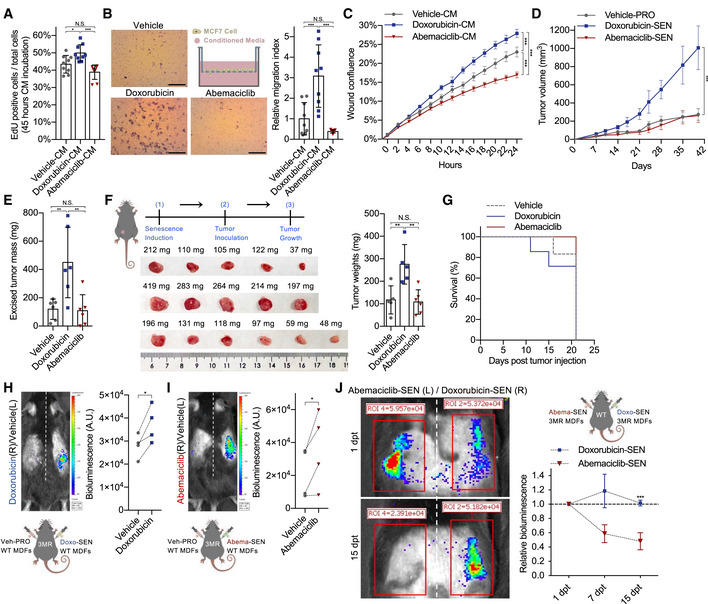
CDK4/6i‐induced senescence lacks pro‐tumorigenic properties but retain ability to promote paracrine senescence and undergo clearance AMCF7 cells were incubated with serum‐free CM collected from treated BJ fibroblasts (8 dpt) containing EdU for 45 h and EdU^+^ cells quantified (*n* = 9 samples from 3 independent experiments).BMCF7 cells migrated through the pores of transwell after 24 h of incubation with CM (8 dpt; scale bar, 1 mm; *n* = 9 samples from 3 independent experiments) were stained with 0.2% crystal violet and quantified.CMigration of A549 cells exposed to CM collected from treated BJ cells (8 dpt; *n* = 4 independent samples, imaged together) was evaluated using a scratch assay. Imaging and analysis were done using a live imaging system.D, ETreated BJ fibroblasts (2.5*10^5^) were co‐injected with A549 cancer cells (10^6^) subcutaneously in *Foxn1^Nu^* mice, tumor growth was measured at the indicated times (*n* = 6 mice/group) (D), and the excised tumors were weighed (E).F, Gp16‐3MR mice were treated with vehicle (PBS, 7 consecutive days), doxorubicin (5 mg/kg, 3 consecutive days), or abemaciclib (50 mg/kg, 7 consecutive days). *n* = 6 or 7 mice/group. At 14 dpt, MMTV‐PyMT mouse breast cancer cells were implanted in the mammary fat pad of treated female mice. The tumors were excised and weighed 21 days post‐inoculation (left panel, images of the tumors; right panel, tumor weights) (F). The survival curves for different groups were plotted (G).H, I2 × 10^5^ cells of vehicle‐treated (water for 8 times in 24 h) mouse dermal fibroblasts (MDFs) were injected into the left flank of the p16‐3MR mice. Doxorubicin‐induced (250 nM for 24 h) (H) or abemaciclib‐induced (4 μM for 8 times in 24 h) (I) MDFs were injected into the right flank of the same animal. 7 dpt, the above‐mentioned mice were injected with coelenterazine and bioluminescence from the p16‐3MR mouse was visualized/quantified by the IVIS spectrum *in vivo* imaging system and quantified. Lower panel, scheme of experimental design (*n* = 4 mice/group).JEqual amount (2 × 10^5^ cells) of doxorubicin‐induced (250 nM for 24 h, right flank) and abemaciclib‐induced (4 μM for 8 times in 24 h, left flank) senescent MDF‐3MR cells was subcutaneously injected into the same wild‐type mice. At 1 dpt, 7 dpt, and 15 dpt, the above‐mentioned mice were injected with coelenterazine and bioluminescence from the injected MDF‐3MR cells was visualized/quantified by the IVIS spectrum *in vivo* imaging system and quantified (*n* = 4 mice/group). Upper panel, scheme of experimental design. Data information: Data are means ± SD. One‐way ANOVA (A, B, E, and F). Two‐way ANOVA (C, D and J). Paired Student’s *t*‐test (H and I). *P < 0.05, **P < 0.01, and ***P < 0.001, N.S. = not significant. dpt, days post‐treatment. PRO, proliferating cells. SEN, senescent cells. CM, conditioned media.

**Figure EV5:**
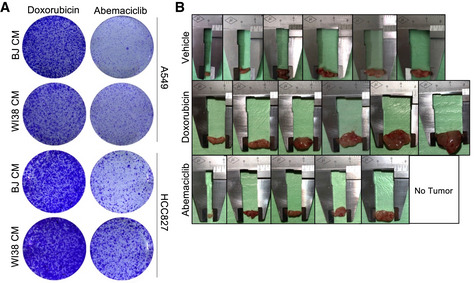
p53‐associated SASP lacks pro‐tumorigenic properties A549 and HCC827 lung cancer cells were incubated with drug‐induced BJ or WI38 serum‐free CM for 45 h. Cells were replated for colony formation assay for 8 days (*n* = 3 independent experiments).Excised tumors from Fig 5D (*n* = 6 mice/group).

NF‐kB‐associated secretory phenotype factors can enhance cancer cell proliferation and migration (Laberge *et al*, 2015a). To understand whether CDK4/6i‐induced senescent cells—which express a PASP but lacks the NASP—influence cancer cell proliferation, we measured the growth of MCF7 (breast cancer) (Fig 5A), and A549 and HCC827 (lung cancer) (Fig EV5A) cells exposed to the conditioned media (CM) of doxorubicin‐induced or abemaciclib‐induced senescent cells. For all cells, the CM of doxorubicin‐induced senescent cells accelerated proliferation, but the CM from abemaciclib‐induced senescence did not increase cancer cell growth (Fig EV5A). Then, we used a similar system to measure cancer cell migration. Again, a favorable effect on migration was observed for MCF7 and A549 cells exposed to the CM of doxorubicin‐induced senescent BJ fibroblasts, while no effects were measured in cells exposed to the CM abemaciclib‐induced cells (Fig 5B and C). We then co‐injected doxorubicin‐ or abemaciclib‐induced senescent BJ and lung cancer cells (A549) into the flanks of *Foxn1* ^Nu^ mice. Similar to the observations made in cell culture, only the presence of doxorubicin‐induced, but not of abemaciclib‐induced, senescent cells promoted tumorigenesis (Figs 5D and E, and EV5B). At last, we repeated a similar experiment using an orthotopic model. The murine breast carcinoma cells MMTV‐PyMT were transplanted in C57BL/6 mice pretreated with either doxorubicin or abemaciclib. Mice pretreated with doxorubicin showed accelerated tumor growth (Fig 5F) and reduced survival rate (Fig 5G), while mice treated with abemaciclib did not show significant differences when compared to control animals.

Cellular senescence can exert tumor suppressive functions via non‐cell‐autonomous mechanisms, namely by inducing paracrine senescence and promoting immunosurveillance and their own immune‐mediated clearance. To evaluate paracrine senescence, we injected wild‐type senescent MDFs induced to senescence by doxorubicin or abemaciclib into the dorsal skin of p16‐3MR mice. Interestingly, both subsets of senescent cells were equally able to promote luminescence in the transplanted area of the recipient mice, suggesting induction of p16^+^ cells (Fig 5H and I). Importantly, luminescence was not promoted by injection of non‐senescent MDFs, confirming the validity of this *in vivo* assay for measuring paracrine senescence (Fig 5H and I). One of the major drivers of detrimental functions of senescent cells is their impaired turnover due to higher induction rates and lower immune‐mediated clearance. No significant differences in the induction and accumulation of senescence were observed between the abemaciclib and doxorubicin groups at the time points chosen for phenotypical analyses (Fig 2A–E). To better characterize clearance rates, we decided to use a transplantation system that could compare removal of different subsets of senescent cells in the same immunological environment. MDFs derived from the p16‐3MR mouse were induced to senescence *ex vivo* with doxorubicin or abemaciclib, and then transplanted into opposite flanks of immunocompetent and syngeneic wild‐type untreated mice (Fig 5J). Unexpectedly, the absence of the NASP did not delay clearance, and abemaciclib‐induced senescent cells showed even faster removal compared to the doxorubicin‐treated cells (Fig 5J). Overall, our data show that CDK4/6i‐induced senescence, expressing a unique SASP free of NF‐κB‐driven but enriched in p53‐associated SASP factors, does not acquire pro‐tumorigenic properties while retaining the ability to promote paracrine senescence and facilitate clearance.

## Discussion

Collectively, we show here that normal cells treated with CDK4/6 inhibitors (CDK4/6i) enter a program of stable growth arrest in which establishment is dependent on the transcriptional activity of the tumor suppressor p53. Normal cells without functional p53 activate a partial senescent phenotype without permanent growth arrest, suggesting that intact p53 functions are necessary for a complete response to CDK4/6 inhibition. While non‐cancerous cells are likely to have wild‐type and functional p53, p53 mutations are observed in > 50% of human malignancies (Muller & Vousden, 2013). Interestingly, previous studies have reported that breast cancer patients carrying p53 mutations are more likely to respond poorly to CDK4/6i (Patnaik *et al*, 2016), and that breast cancer cells with wild‐type p53 are more sensitive to radiotherapy (Fernández‐Aroca *et al*, 2019). Moreover, it was shown that malignant transformation of hematopoietic cells depends on CDK6‐mediated repression of p53 activity and that CDK6 inhibition could reverse this effect (Bellutti *et al*, 2018). However, the genomic landscape of cancer cells remains highly heterogeneous and unstable, making the demonstration of a direct correlation between p53 activity and irreversible cell cycle arrest in tumors a challenging task. In our non‐transformed models, enhanced p53 activity could be related to increased mitochondrial ROS production. However, higher mitochondrial ROS production was not a cause or consequence of elevated DNA damage, as DDR signaling in abemaciclib‐induced senescence remained low. Interestingly, previous reports have shown that exposure to CDK4/6i leads to epigenetic changes potentially favoring accessibility of p53 to its target genes (Acevedo *et al*, 2016; Goel *et al*, 2017). Thus, future studies should aim at understanding the contribution of both inducers of p53 activity and regulators of accessibility to p53 direct targets in cells exposed to CDK4/6i.

We also show that CDK4/6i‐treated normal cells enter a senescent program characterized by morphological changes, suppression of many cell cycle‐related genes, induction of a core senescence‐associated signature, and activation of lysosomal content and enzymes, but lacking many of the common pro‐inflammatory SASP factors. This last observation is in agreement with previous reports showing that genetic overexpression of the endogenous CDK4/6i p16 leads to a senescent state with lower SASP (Coppé *et al*, 2011). However, recent reports have highlighted those differences in SASP expression might not be only quantitative but also qualitative (Hoare *et al*, 2016; Wiley *et al*, 2016).

Indeed, SASP composition might be dependent on various factors, including tissue, cell type, and time point, and promoted by various signaling pathways, including NF‐κB, mTOR, cEBPβ, and p38 (Coppé *et al*, 2010; Freund *et al*, 2010; Laberge *et al*, 2015b). In contrast, p53 was shown to interfere with the induction of SASP programs, in particular via inhibition of NF‐κB (Wiley *et al*, 2018) and mTOR (Hasty *et al*, 2012) signaling. However, during the characterization of the CDK4/6i‐induced senescence, we were able to identify a SASP transcriptional program that is specifically promoted by p53 activity—a phenotype named PASP. Treatment with CDK4/6i induces a PASP but not a NF‐κB‐associated secretory phenotype (NASP), reflecting the high p53 transcriptional activity but the lack of chronic DDR and activation of NF‐κB signaling in cells exposed to this treatment (Rodier *et al*, 2009; Chien *et al*, 2011). Prolonged abemaciclib exposure causes premature senescence *in vivo*, but the abemaciclib‐induced senescent cells were well tolerated, suggesting that PASP genes do not exert detrimental functions. These data fit with the notion that toxicity of CDK4/6i in patients is generally lower than upon treatment with standard chemotherapy (Klein *et al*, 2018), and suggest that CDK4/6i could be better tolerated by cancer populations that are at high risk of developing side effects. Moreover, the presence *in vivo* of p16^+^ senescent cells without NASP factors and detrimental effects emphasizes how heterogeneous the senescence phenotype can be, and the importance of carefully phenotyping senescent cells to determine their potential toxicities. According to our data, one of the regulators of senescence‐associated toxicities is predicted to be the NASP, while senescent cells with only a PASP might be tolerated and potentially less dangerous. Thus, we suggest that a subset of NASP and PASP factors should be used as biomarkers to determine the detrimental function of senescent cells *in vivo*, and also as readouts for senotherapies. In the future, it will be essential to also understand how the different SASP modules integrate and influence each other. In particular, because cells with PASP only were cleared at a faster rate, it will be interesting to identify PASP factors that facilitate, and NASP factors that interfere with, immune responses (Ventura *et al*, 2007; Xue *et al*, 2007; Iannello & Raulet, 2013; Stokes *et al*, 2019).

## Materials and Methods

### Cell culture and drug administration

BJ (CRL‐2522), WI38 (CRL‐7728), MMTV‐PyMT (CRL‐3278), A549 (CCL‐185), HCC827 (CRL‐2868), MCF7 (HTB‐22), and hTERT‐RPE1 (CRL‐4000) cells were purchased from ATCC. Primary human MSCs were a gift from Prof. Irene Heijink (University Medical Center Groningen, The Netherlands). MEFs were generated from 13.5‐day wild‐type embryos. MDFs were isolated from the dorsal skin of 3‐month‐old p53‐null mice or wild‐type littermates and a gift from Prof. Paul Hasty (University of Texas Health Science Center at San Antonio, USA). Cells were not re‐authenticated by the laboratory but were regularly monitored for mycoplasma contaminations (once per month). No cell line used was listed in the database of commonly misidentified cell lines maintained by ICLAC. All cells were cultured in DMEM–GlutaMAX (Thermo Fisher) medium supplemented with 10% fetal bovine serum (GE Healthcare Life Sciences) and 1% penicillin–streptomycin (Lonza). All the human and mouse normal primary cells were maintained in 5% O_2_, 5% CO_2_, and 37°C incubators, and all cancer cells were maintained in 20% O_2_, 5% CO_2_, and 37°C incubators. For drug treatment experiments, cells were plated in 6‐well dishes with 3 technical repeats (3 × 10^4^ cells/well, around 30% confluence). Doxorubicin hydrochloride (Tebu‐bio, BIA‐D1202‐1) was dissolved in sterile Milli‐Q water at 250 μM as stock. Paclitaxel (MedChemExpress, HY‐B0015) was dissolved in DMSO at 10 mM as stock. Palbociclib isethionate (Sigma‐Aldrich, PZ0199) was dissolved in sterile Milli‐Q water at 50 mM as stock. Abemaciclib (MedChemExpress, HY‐16297) was dissolved in sterile Milli‐Q water at 50 mM as stock. Nutlin‐3a (MedChemExpress, HY‐10029) was dissolved in DMSO at 10 mM as stock. Pifithrin‐α hydrobromide (MedChemExpress, HY‐15484) was dissolved in DMSO at 10 mM as stock. All the drugs were further diluted in above‐mentioned DMEM to treat cells at different concentrations as indicated in each figure legend.

### Colony formation assay

Drug‐treated normal or breast cancer cells were replated in a 6‐well dish (3 × 10^3^ cells/well) at the end of treatment and allowed to grow in drug‐free normal medium for 8 days. Cancer cells were plated in a 6‐well dish (3 × 10^4^ cells/well) and incubated with serum‐free conditioned media (CM) for 45 h. Afterward, cells were fixed in 4% PFA for 30 min and then stained with 0.2% crystal violet in 37% methanol for 1 h. Pictures of all the plates were taken with a scanner (Epson). The images were cropped and processed in Microsoft PowerPoint using the same settings.

### EdU staining

Drug‐treated cells were replated on coverslip in a 24‐well plate (3 × 10^4^ cells/well) and cultured for 10 or 20 h in the presence of EdU (10 μM), then fixed and stained as previously described (Hernandez‐Segura *et al*, 2018a). Images were acquired at 100 times magnification (Leica), and the number of cells was counted with the software ImageJ. The images were cropped using Microsoft PowerPoint.

### Senescence‐associated β‐galactosidase staining

For staining of cells, drug‐treated cells were replated in a 24‐well plate (2 × 10^4^ cells/well), and the SA‐β‐galactosidase staining was done as described previously (Hernandez‐Segura *et al*, 2018a). For *in vivo* kidney section staining, kidneys from drug‐treated and control animals were immediately imbedded in O.C.T (Sakura, 621232), snap‐frozen in liquid nitrogen, and stored in −80°C. The sectioning (10 μm) was done using cryostat 1 h prior to staining, and the slides were washed with prechilled PBS on ice in a glass Coplin jar twice for 5 min and fixed in a mixture of formaldehyde (2%) and glutaraldehyde (0.2%) for 10 min on ice. After fixation, the slides were washed briefly with Milli‐Q water, and then stained with the staining buffer overnight in 37°C incubator without CO_2_. The staining buffer included 1 mg/ml X‐gal (Thermo Fisher) in dimethylformamide, 40 mM citric acid/Na phosphate buffer, 5 mM potassium ferrocyanide, 5 mM potassium ferricyanide, 150 mM sodium chloride, and 2 mM magnesium chloride. Next day, the staining solution was washed away with Milli‐Q water and the slides were mounted with 70% glycerol. Images were acquired at 100× magnification with a microscope (Leica) and were processed in Microsoft PowerPoint. The SA‐β‐gal‐positive area and total area were measured and quantified by with the software ImageJ.

### Conditioned media (CM) collection and analyses

BJ fibroblasts were treated with vehicle (water—8 times in 24 h; 1 in 1,000), doxorubicin (250 nM—1 time 24 h), or abemaciclib (1 μM—8 times in 24 h). After the treatments, cells were cultured with drug‐free normal medium for 8 days. Thereafter, cells from different groups were trypsinized and counted, and 10^6^ cells from each group were replated and incubated with serum‐free medium for 24 h. Then, the CM was collected and centrifuged at 300 g/5 min to remove any floating cell or debris. The CM was kept in −80°C after harvesting and thawed on ice for further analyses or experiments. Human Cytokine Antibody Array (Abcam, ab133997) was used following the manufacturer’s instructions. After the images were taken, density of positive controls from 3 membranes was equalized using Adobe Photoshop, and individual factors from different membranes were compared. High contrast was adjusted to highlight the most interesting SASP factors and to visualize the lowly expressed factors. For ELISA, human CXCL1/GROα duo‐set or CCL5 duo‐set or IGFBP3 duo‐set (R&D Systems) was used to detect the concentrations of CXCL1 or CCL5 or IGFBP3 in the CM following the manufacturer’s instructions.

### Western blot

Total cell lysates were obtained by resuspension of the cells in RIPA buffer (cat# ab156034, Abcam) supplemented with proteinase and phosphatase inhibitors (cat# A32959, Pierce (TFS)). Protein concentration was measured using a BCA protein assay kit (cat# 10741395, Pierce (TFS)), and subsequently, protein sample buffer was added to a final concentration of 40 mM Tris–HCl (pH 6.8), 1.9% SDS, 3.1% β‐mercaptoethanol, and 6,3% glycerol. Samples were boiled for 5 min. Equal amount of proteins was loaded onto 12% SDS–PAGE (acrylamide: bis‐acrylamide 29:1, cat# 161‐0146, Bio‐Rad) and after size separation blotted onto 0.2‐µm nitrocellulose membrane (cat# 162‐0112, Bio‐Rad). Immunodetection was performed by standard procedures of p16/Ink4a (clone EPR1473, cat# ab108349, Abcam), p21 (clone C‐19, cat# sc‐397, Santa Cruz Biotechnology), p53 (clone DO‐1, cat# sc‐126, Santa Cruz Biotechnology), Rb (clone 4H1, cat# 9309, Cell Signaling Technology), and phospho‐Rb (ser795, cat# 9301, Cell Signaling Technology). The antibodies for the proteins of interest were used in 1:1,000 dilution in 5% milk/TBST (Tris‐buffered saline with 0.1% Tween‐20) and incubated overnight at 4°C. Immunodetection of vinculin (cat# V9131, Sigma‐Aldrich) or β‐Actin (clone C4, cat# 08691001, MP Biomedicals) or Lamin A/C (Santa Cruz, sc‐71481) was performed as loading control. ECL Prime Western Blotting Detection Reagent (cat# RPN2232, GE Healthcare) was used according to the manufacturer’s guidelines for detection, and the signal was measured using an ImageQuant LAS 4000 biomolecular imager (GE Healthcare). Densitometry was performed using ImageJ software (NIH—Public domain), and values were corrected for protein input as measured by the loading control.

### Generation of a lentiviral NF‐κB reporter vector

Plasmid pGL4.32[luc2P/NF‐κB‐RE/Hygro] (cat# E8491, Promega) was used as template DNA in a PCR for obtaining the minimal promoter Luc2P together with the NF‐κB response elements (NF‐κB‐RE). The DNA polymerase used was Phusion High Fidelity (cat# E0553S, New England Biolabs (NEB)): forward primer: 5'‐aaaaATCGATgg cctaactggccggtacc‐3’, reverse primer 5'‐aaaaGGATCCcgactctagagtcgcggcc‐3'. The PCR product was purified from gel cut with ClaI (cat#10656291001, Roche) and BamHI‐HF (cat# R3136s, NEB) overnight at 16°C. This fragment was ligated into the backbone vector pLenti CMV GFP Hygro (656‐4) (restriction with ClaI and BamHI‐HF released the CMV promoter from the lentiviral backbone sequence). As previously described in Vliet *et al* (2021), pLenti[CMV/GFP/Hygro] (656‐4) was a gift from Eric Campeau & Paul Kaufman (Addgene plasmid #17446). The cut backbone vector was treated with Antarctic phosphatase (cat# M0289S, NEB) before ligation. T4 DNA ligase (cat# M0202S, NEB) was used in the ligation reaction. All enzymes were used according to the manufacturer’s manual. The ligation reaction products were transformed into NEB stable competent *E. coli* (high efficiency, cat# c3040h, NEB) and grown overnight at 37°C on LB plates supplemented with 50 µg/ml ampicillin. A colony PCR was performed to check for positive clones. Single positive clones were grown overnight in liquid LB supplemented with ampicillin at 37°C to be midiprepped the next day using a PureLink^®^ HiPure Plasmid Midiprep Kit (cat# K210005, Invitrogen). This newly generated lentiviral NF‐κB‐reporter vector (pLenti‐NF‐κB‐RE‐minP‐LUC‐EGFP‐hygro) was sent to GATC/Eurofins for sequencing and verification of the Luc2P/ NF‐κB‐RE insert. Recombinant human TNF‐α (PeproTech, 300‐01A) (10 ng/μl; 6 h treatment) was used as positive control to validate the NF‐κB reporter.

### Generation of a lentiviral p53 reporter vector

Plasmid PG13‐luc (wt p53 binding sites) was used as template DNA in a PCR for obtaining the p53 wild‐type binding sites together with the luciferase reporter. PG13‐luc (wt p53 binding sites) was a gift from Bert Vogelstein (Addgene plasmid # 16442). The DNA polymerase used was Phusion High Fidelity (cat# E0553S, NEB): forward primer 5'‐aaaaaGGGACCCaaaCGATAA GCTTGATGCC‐3’, reverse primer 5'‐tttttGTCGACaaaTTAAATCTC TGTAGG‐3'. The PCR product was purified from gel and cut with KflI (cat# FD2164, Thermo Fisher Scientific) and SalI (cat# R3138, NEB) for 15 min at 37°C. This cut PCR product was ligated into the backbone vector pLenti CMV GFP Hygro (656‐4) (restriction with KflI and SalI releases the cPPT/CST element, the CMV promoter, and the EGFP from the lentiviral backbone sequence). As previously described in Vliet *et al* (2021), the cut backbone vector was treated with Antarctic phosphatase (cat# M0289S, NEB) before ligation. T4 DNA ligase (cat# M0202S, NEB) was used in the ligation reaction. All enzymes were used according to the manufacturer’s manual. The ligation reaction products were transformed into NEB stable competent *E. coli* (high efficiency, cat# c3040h, NEB) and grown overnight at 37°C on LB plates supplemented with 50 µg/ml ampicillin. A colony PCR was performed to check for positive clones. Single positive clones were grown overnight in liquid LB supplemented with ampicillin at 37°C to be midiprepped the next day using a PureLink^®^ HiPure Plasmid Midiprep Kit (cat# K210005, Invitrogen). This newly generated intermediate lentiviral p53 reporter vector (pLenti–p53 WT bind.sites‐LUC‐hygro) was still dysfunctional because of the lack of cPPT/CST element. The cPPT/CST element has been repaired before the newly generated lentiviral p53 reporter vector (pLenti–p53 WT bind.sites‐LUC‐hygro) was sent to GATC/Eurofins for sequencing and verification of the cPPT/CST‐p53 WT bind.sites‐LUC insert. Nutlin‐3a (MedChemExpress, HY‐10029) (10 µM; 24‐h treatment) was used as positive control to validate the p53 reporter.

### Production of lentivirus

As previously described in Vliet *et al* (2021), 293FT cells were seeded in a 10‐cm petri dish at 6 × 10^6^ cells. Next day, cells were transfected with the ViraPower plasmid mix (cat# K4970‐00, Invitrogen) together with the lentiviral vectors using PolyFect Transfection Reagent (cat# 301105, Qiagen) overnight. The medium with transfection mix was replaced for normal growth medium without antibiotics, collected after 24 h, and concentrated using Peg‐It Virus precipitation solution (cat# LV810A‐1, System Biosciences) according to the manufacturer’s manual.

### Lentivirus transduction

Lentiviral particles were either generated by the laboratory (NF‐κB or p53 reporter) or obtained from the MISSION shRNA library (Sigma‐Aldrich). These obtained lentiviral particles harbor the following shRNA clones in pLKO.1 backbone vector and were used to infect BJ cells: p16 (*CDKN2A‐INK4A*)–TRCN0000255853; p53 (*TP53*)–TRCN0000003755. Titers of these lentiviral particles were all equal to or higher than 1.8E+07 TU/ml. 10^5^ BJ cells per well were seeded in a 6‐well plate format on Day 0. On Day 1, cells were infected with the lentiviral particles (1:20 dilution in complete medium) for 24 h. Polybrene (6 µg/ml; cat# sc‐134220, Santa Cruz Biotechnology) was added together with the lentiviral particles to enhance the infection efficiency. Cells were washed and refreshed with complete medium on Day 2. Selection with puromycin (2 µg/ml; cat# P8833, Sigma‐Aldrich) started on Day 3 for 3 days. BJ cells infected with pLKO.1‐scrambled lentiviral particles were used to serve as control cells.

### RNA‐sequencing assay

Total RNA was extracted from treated cells via an ISOLATE II RNA Mini Kit (Bioline, cat# BIO‐52073) following the manufacturer’s instructions. The extracted RNA was quantitated using a Nanodrop, and RNA quality was measured via BioAnalyzer RNA Chip (Agilent). Poly‐A tail selection was used to enrich for messenger RNA (mRNA) using the NEXTflex Poly(A) Beads Kit (Bioo Scientific Corp, cat# 512980). RNA‐Seq library preparation was carried out using NEXTflex Rapid Directional qRNA‐Seq Kit (Bioo Scientific, cat# 5130‐05D). In brief, mRNA was fragmented using a cationic buffer and then submitted to first and second strand synthesis, followed by adenylation, ligation, and 15 cycles of PCR. Library quality and size distribution were validated on a Qubit (Thermo Fisher Scientific) and an Agilent High Sensitivity DNA chip. Clusters for sequencing were generated on the cBot (Illumina). Paired‐end sequencing was performed at 400 M reads per lane of the flow cell on an Illumina HiSeq 2500. Average quality scores for the completed run across all samples were > 30, with an average of 10 million reads for each pooled sample. The read length was 76 bp. Raw sequencing data were demultiplexed according to library‐specific barcodes and converted to fastq format using standard Illumina software (bcl2fastq version 1.8.4). The resulting reads were mapped to the human reference genome (GRCh38) using Bowtie2 (version 2.2.4). Sequencing data are available on ArrayExpress under accession no. E‐MTAB‐7642. For differential gene expression, we used DESeq2 (Love *et al*, 2014) to evaluate the genes that were differentially expressed among all the different treatments compared with the vehicle‐treated control. We considered those genes with an adjusted *P*‐value lower than 0.01 as being differentially expressed. A profile of senescence‐associated secretory phenotype (SASP) genes was generated based on literature and previous knowledge, detailed information provided as in Table EV2. GraphPad Prism 7 was used to generate the heatmap using the logarithm base 2 of fold change. Enriched pathways in the differentially expressed genes were evaluated using the online tool “Over‐representation analysis” of the Consensus Path DB‐human (http://cpdb.molgen.mpg.de/) (Kamburov *et al*, 2013). For the Venn plots showing similarly expressed genes in different conditions, only genes that were differentially expressed with a fold change in the same direction (up‐ or downregulated in samples compared) were considered. Venn plots were generated in R version 3.5.1 using the packages “VennDiagram”.

### qRT–PCR assay

Total RNA was isolated using an Isolate II RNA Mini Kit (Bioline). 250–500 ng of RNA was reverse‐transcribed into cDNA using the High‐Capacity cDNA Reverse Transcription Kit (Applied Biosystems). qRT–PCRs were performed using the Universal Probe Library System (Roche) and a SensiFAST Probe Kit (Bioline). Expression of tubulin was used to normalize the expression of target genes. Every biological replicate was analyzed in duplicate. List of primers is provided in Table EV3.

### Chromatin immunoprecipitation (ChIP) assay

BJ cells treated with vehicle, abemaciclib (1 μM—8 times in 24 h), or nutlin‐3a (10 μM—8 times in 24 h), or doxorubicin (250 nM for 24 h) were crosslinked and processed using the ChIP kit (ab500, Abcam) following the manufacturer’s instructions. 6 rounds of sonication (high energy, 30 seconds on/30 seconds off) were done using a sonicator. After DNA was purified, qRT–PCR was done with SYBR Green SuperMix (1725120, Bio‐Rad) using primers such as *CDKN1A#1* (forward primer*—*GGAGGCAAAAGTCCTGTGTTC; reverse primer*—*GGAAGGAGGGAATTGGAG AG), *CDKN1A#2* (forward primer—GAAATGCCTGAAAGCAGAGG; reverse primer—GCTCAGAGTCTGGAAATCTC), *CDKN1A#3* (forward primer—CACCACTGAGCCTTCCT CAC; reverse primer—CTGACTCCCAGCACACACTC), *CDKN1A#4* (forward primer—GATGCCAACCAGATTTGCCG; reverse primer—CCTGGCTCTAACAACATCCC), *MDM2* (forward primer—GGTTGACTCAGCTTTTCCTCTTG; reverse primer—GGAAAATGCA TGGTTTAAATAGCC), *GADD45A* (forward primer—AGCGGAAGAGATCCCTGTGA; reverse primer—CGGGAGGCAGGCAGATG), and *IGFBP3* (forward primer—CGAGCCC CTGAGGCAAA; reverse primer—GCTTGGTGTCCAGCTCAGATG).

### Transwell migration assay

As previously described in Vliet *et al* (2021), MCF7 breast cancer cells (2 × 10^4^) were resuspended in serum‐free medium and seeded in a transwell insert (8 μm pore, Corning) with serum‐free media collected from drug‐induced senescent fibroblasts in the outer chamber of a 24‐well culture dish. Cells were cultured in a 5% O_2_, 5% CO_2_, and 37°C incubator for 16 h. Thereafter, cells migrated to the lower side of the transwell were fixed with 4% PFA and stained with 0.2% crystal violet. Images were acquired at 40× magnification with a microscope (Leica).

### Scratch migration assay and IncuCyte imaging

A549 lung cancer cells (4 × 10^4^) were plated in a 96‐well ImageLock plate (Sartorius) and cultured in a 20% O_2_, 5% CO_2_, and 37°C incubator for 24 h. Then, the 96‐pin IncuCyte WoundMaker was used to create precise and reproducible cell‐free zone in each well. Cells were washed with PBS and then incubated with serum‐free conditioned media collected from drug‐induced senescent fibroblasts for 24 h in the IncuCyte imaging machine. Images were taken every 2 h and analyzed by the IncuCyte software.

### Detection of mitochondrial ROS

BJ cells were treated with vehicle or abemaciclib (1 μM ‐ 4 times in 24 h) and harvested for the MitoROS (AAT Bioquest, 16052) staining. The cells were stained for 10 min at 37°C in darkness. Following the staining, cells were centrifuged at 400 g for 5 min and cell pellet was resuspended in serum‐free DMEM for analysis by flow cytometry.

### *In vivo* animal experiments

All the mice were maintained in the central animal facility (CDP) of University Medical Center Groningen (UMCG) under standard conditions. All the experiments were approved by the Central Authority for Scientific Procedures on Animals (CCD—License #AVD105002015339 and #AVD1050020184807 and #AVD10500202114482) in the Netherlands.

For abemaciclib anti‐tumor effect validation, 10^6^ fLUC‐MMTV‐PyMT cancer cells, previously generated (Demaria *et al*, 2017), were injected into the mammary fat pad of 13‐week‐old female p16‐3MR mice under anesthesia (10 mice). At 7 days and 14 days post‐cell injection, the mice were injected with 150 mg/kg D‐Luciferin (SanBio, 14681‐1). 8 min later, the mice were anesthetized with 2% isoflurane and firefly bioluminescence was visualized/quantified using the IVIS Spectrum *In Vivo* Imaging System (PerkinElmer, 5‐minute medium binning) in CDP‐UMCG. The mice were equally distributed in two groups based on tumor size (bioluminescence). The female mice bearing breast cancer were then injected i.p. with vehicle (PBS, 7 consecutive days) or abemaciclib (in PBS, 50 mg/kg, 7 consecutive days) (*n* = 5 mice/group). After the second bioluminescence imaging, the mice were terminated, and tumors were excised for weighing.

For senescence induction and healthspan analysis in p16‐3MR, both male and female mice were used. 14‐week‐old healthy mice were injected i.p. with vehicle (PBS, 7 consecutive days), doxorubicin (in PBS, 5 mg/kg, 3 consecutive days), or abemaciclib (in PBS, 50 mg/kg, 7 consecutive days) (*n* = 6 mice/group). All the treatments were finished at the same day. At 14 days after above‐mentioned treatments, the mice were injected with 100 μl Xenolight RediJect Coelenterazine h (PerkinElmer, 760506). 20 min post‐injection, mice were anesthetized with 2% isoflurane and Renilla bioluminescence was visualized/quantified by the IVIS Spectrum *In Vivo* Imaging System (PerkinElmer, 5‐minute medium binning) in CDP‐UMCG. Forelimb grip strength (N) was measured using a grip strength meter (Columbus Instruments) at 7 and 14 days post‐treatments, and results were averaged from 5 trials. Hanging tests were performed at 7 and 14 days post‐treatments. The mice were placed under the grid of above‐mentioned grip strength meter with the bedding below to protect the mice when they fell down. A cut‐off time of 300 s was determined, and the hanging time was normalized to body weights as hanging duration (sec) multiplied by body weight (g). At 15 days post‐treatments, endurance tests were performed on an accelerating RotaRod (IITC Life Science) using a top speed of 40 rpm over a period of 300 s. Each mouse was put on the machine to adapt twice; four hours later, two trials were recorded for each mouse. The average of the two trials was calculated and plotted. For *ex vivo* analysis, kidneys and livers were both snap‐frozen in liquid nitrogen for RNA/protein isolation and imbedded in O.C.T (Sakura, 621232) (frozen in liquid nitrogen) for cryosectioning. All the samples were stored in −80 °C until analyzed. 20 μg total protein was collected from each treated kidney, and IL‐6 protein level was determined by the mouse IL‐6 duo‐set (R&D Systems) ELISA. The experiments were done following the manufacturer’s instructions. Part of the plasma was used for *in vivo* ELISA, and 100 μl plasma from each mouse was tested using the mouse CXCL1/KC duo‐set (R&D Systems). The experiments were done following the manufacturer’s instructions. Around 25 μl of plasma was diluted and used for red blood cell and white blood cell counting (Medonic CA620). Around 900 μl of plasma from each mouse was used to stain for T cell (1:150, APC‐CD3e/clone 145‐2C11, Biolegend cat# 100312), B cell (1:300, FITC‐CD45R/B220/clone RA3‐6B2, Biolegend cat# 103205), and granulocyte and macrophage (1:1000, PECy7‐Gr‐1/clone RB6‐8C5 and PECy7‐CD11b /clone M1/70, Biolegend cat# 108416 and cat# 101216), then analyzed by flow cytometry (BD MoFlo XDP).

For RFP^+^ cells sorting from above‐mentioned doxorubicin‐ and abemaciclib‐treated mice, the cortex of mouse kidneys was physically separated from the medulla and dissociated first mechanically and later enzymatically in a Collagenase A 4 mg/ml (Roche, 10103586001) solution in DMEM without FBS supplementation. The tissue was incubated for 30 min at 37°C, with agitation. The homogenized kidney cortex was then filtered through a 70 μm cell strainer (Corning, CLS431751‐50EA) to obtain a single‐cell suspension, and the pellet was washed 3 times with PBS. Cells were resuspended in PBS and incubated with DAPI and Draq5 for 10 min on ice. In a MoFlo Astrios sorter (UMCG‐FCU), viable cells were sorted as DAPI^‐^/Draq5^+^ population. RFP^+^ cells, establishing previously the gating with a WT mouse sample, were sorted from individual mice, and collected in RLY Lysis Buffer (Bioline, Isolate II Rna Micro Kit) for RNA isolation.

For abemaciclib+ pifithrin‐α experiments in p16‐3MR, both male and female mice were used. 14‐week‐old healthy mice were injected i.p. with abemaciclib (in PBS, 50 mg/kg, 7 consecutive days) + vehicle (5% DMSO in PBS) or abemaciclib + pifithrin‐α (5% DMSO in PBS, 2 mg/kg, 7 consecutive days) (*n* = 5 mice/group). All the treatments were finished at the same day. At 20 days after above‐mentioned treatments, the mice were injected with 100 μl Xenolight RediJect Coelenterazine h (PerkinElmer, 760506). 20 min post‐injection, mice were anesthetized with 2% isoflurane and Renilla bioluminescence was visualized/quantified by the IVIS‐100 *In Vivo* Imaging System (PerkinElmer, 5‐minute medium binning) in CDP‐UMCG. For *ex vivo* analysis, tissues were snap‐frozen in liquid nitrogen for RNA/protein isolation. All the samples were stored in −80°C until analyzed.

For MMTV‐PyMT cancer cell injection in doxorubicin/abemaciclib‐treated mice, 14‐week‐old healthy female mice were injected i.p. with vehicle (PBS, 7 consecutive days; *n* = 6 mice), doxorubicin (in PBS, 5 mg/kg, 3 consecutive days; *n* = 7 mice), or abemaciclib (in PBS, 50 mg/kg, 7 consecutive days; *n* = 6 mice). All the treatments were finished at the same day. At 7 days after above‐mentioned treatments, 3*10^5^ fLUC‐MMTV‐PyMT cancer cells were injected into the mammary fat pad of the treated female mice under anesthesia. The tumors were allowed to grow for 21 days. Then, the mice were terminated, and tumors were excised for weighing and photographs.

For human senescent/cancer cells co‐injection in *Foxn1* ^Nu^ mice, BJ fibroblasts were treated with vehicle (water for 8 times in 24 h; 1 in 1,000), doxorubicin (250 nM for 24 h), palbociclib or abemaciclib (both 1 μM for 8 times in 24 h) *in vitro*. At 8 days post‐drug removal, BJ cells (2.5*10^5^) from each group were co‐injected with A549 cancer cells (10^6^) subcutaneously in right flanks of 12‐week‐old female *Foxn1* ^Nu^ mice (The Jackson Laboratory). 7 days after cell injection, tumor volume was calculated twice a week. The tumor length, width, and depth were measured with a caliper, and the investigator was blinded. The mice were terminated when one of the tumors reached the size for humane endpoint (> 1,500 mm^3^). After the tumors were excised, the weight of each tumor was measured and plotted.

### Patient characteristics and plasma analysis

Paired plasma samples before the start of therapy (pretreatment) and after ± 4 weeks (median 29 days) of treatment (on‐treatment) were obtained from 9 metastatic breast cancer patients who received palbociclib treatment and 10 metastatic breast cancer patients who received paclitaxel treatment. Written informed consent was obtained from all patients. Mean age was 55.9 years (SD 10.9) for the palbociclib group and 60.1 years (SD 7.4) for the paclitaxel group. The samples were collected in cell stabilizing blood collection tubes (CellSave or Streck Cell‐Free DNA BCT tubes). After collection, the blood was centrifuged twice (1700 *g* for 10 min and 12,000 *g* for 10 min). Afterward, the plasma was pooled and directly stored in 2‐ml tubes in −80°C until analyzed. For ELISA, human CXCL1/GROα duo‐set or CCL2 duo‐set or CCL5 duo‐set or MMP1 duo‐set or IGFBP3 duo‐set (R&D Systems) was used to detect the concentrations of CXCL1 or CCL2 or CCL5 or MMP1 or IGFBP3 in the plasma following manufacturer’s instructions.

### Statistics

GraphPad Prism 7 was used for the statistical analyses. Detailed information is provided in each figure legend.

## Author contributions

BW and MD conceptualization the study. BW and MD performed data curation. BW, AH‐S, and MD performed formal analysis. MD involved in funding acquisition. BW, MV‐E, SMB, AH‐S, TV, and EMJ contributed to investigation. BW, MV‐E, AH‐S, and MD contributed to methodology. BW and MD contributed to project administration. BW, AH‐S, EMJ, NO, SMW, AJ, and MD provided resources. MD supervised the study. BW, MV‐E, SMB, AH‐S, TV, and MD validated the data. BW and MD performed visualization. BW and MD wrote the original draft. BW, SMW, AJ, NO, and MD wrote, reviewed, and edited the manuscript.

## Supporting information



Expanded View Figures PDF

Table EV1

Table EV2

Table EV3

## Data Availability

The datasets produced in this study are available in the following database: RNA‐seq data: ArrayExpress E‐MTAB‐7642 (http://www.ebi.ac.uk/arrayexpress/experiments/E‐MTAB‐7642).
