# Supplementary material for: Pharmacological CDK4/6 inhibition reveals a p53‐dependent senescent state with restricted toxicity
Source: EMBO J. 2022 Jan 5;41(6):e108946. doi: 10.15252/embj.2021108946 (PMC8922251; doi:10.15252/embj.2021108946)
Supplement: Supplementary file 1 — Expanded View Figures PDF [file EMBJ-41-e108946-s002.pdf]

Expanded View Figures

Figure EV1. Prolonged CDK4/6i treatment induces p53-dependent cellular senescence.

A–C Human fibroblasts (BJ) were treated with vehicle (water for 8 times in 24 h) or abemaciclib (1 μM for 1 or 8 times in 24 h). Protein was isolated from vehicle or abemaciclib-treated cells and immunoblotted for p-Rb, Rb, and actin (A). RNA was isolated from treated cells, and mRNA levels of E2F2 gene (B) and p16 gene (C) were quantified by qPCR relative to tubulin (internal control). *n* = 3 independent experiments.

D–F 8-day population doubling of WI38 cells treated with vehicle, palbociclib, or abemaciclib (both 1 μM for 8 times in 24 h; *n* = 3 independent experiments) (D). At 8 dpt, treated WI38 cells were incubated with EdU for 10 h and stained (scale bar, 150 μm; *n* = 6 samples from 3 independent experiments) (E). 3 × 103 treated WI38 cells were replated in 6-well dish, cultured for 8 days, and stained with 0.2% crystal violet (*n* = 3 independent experiments) (F).

G, H Cells were treated with vehicle or abemaciclib (1 μM for 1 or 4 or 8 times in 24 h), after drug withdraw, either replated for colony formation assay (*n* = 3 independent experiments) (G) or incubated with EdU for 10 h, and stained at 8 dpt (*n* = 9 samples from 3 independent experiments) (H).

I Cells were treated with vehicle or abemaciclib (250 nM or 500 nM or 1 μM for 8 times in 24 h); after drug withdraw, the cells were replated for colony formation assay (*n* = 3 independent experiments).

J Representative phase-contrast images of BJ or WI38 cells at the end of each drug treatment (scale bar, 1 mm; *n* = 3 independent experiments).

K At 8 dpt, treated BJ cells were fixed and stained for SA-β-gal and quantified (scale bar, 1 mm; *n* = 3 independent experiments).

L Whole-cell lysate of treated BJ cells was used to immunoblot for p16 (*n* = 3 independent experiments).

M–O RNA-sequencing was performed with human fibroblasts (BJ) treated with vehicle (water for 8 times in 24 h) or abemaciclib (1 μM for 8 times in 24 h) (*n* = 3 independent samples, sequenced together). Heatmap of cell cycle genes (M), senescence signature (N), and p53-repressed (red) and p53-activated (blue) cell cycle-related genes (O) calculated from RNA-seq datasets of cells 8 dpt relative to the vehicle-treated group.

P Cells were treated with vehicle or abemaciclib (1 μM for 8 times in 24 h); after drug withdraw, the nuclear fraction was isolated and protein extracted for Western blotting. Lamin A/C was used as the marker of nucleus and loading control (*n* = 3 independent experiments).

Data information: Data are means ± SD. Two-way ANOVA (B, C, and H). One-way ANOVA (D, E, and K). ***$P < 0.001$.

© 2022 The Authors

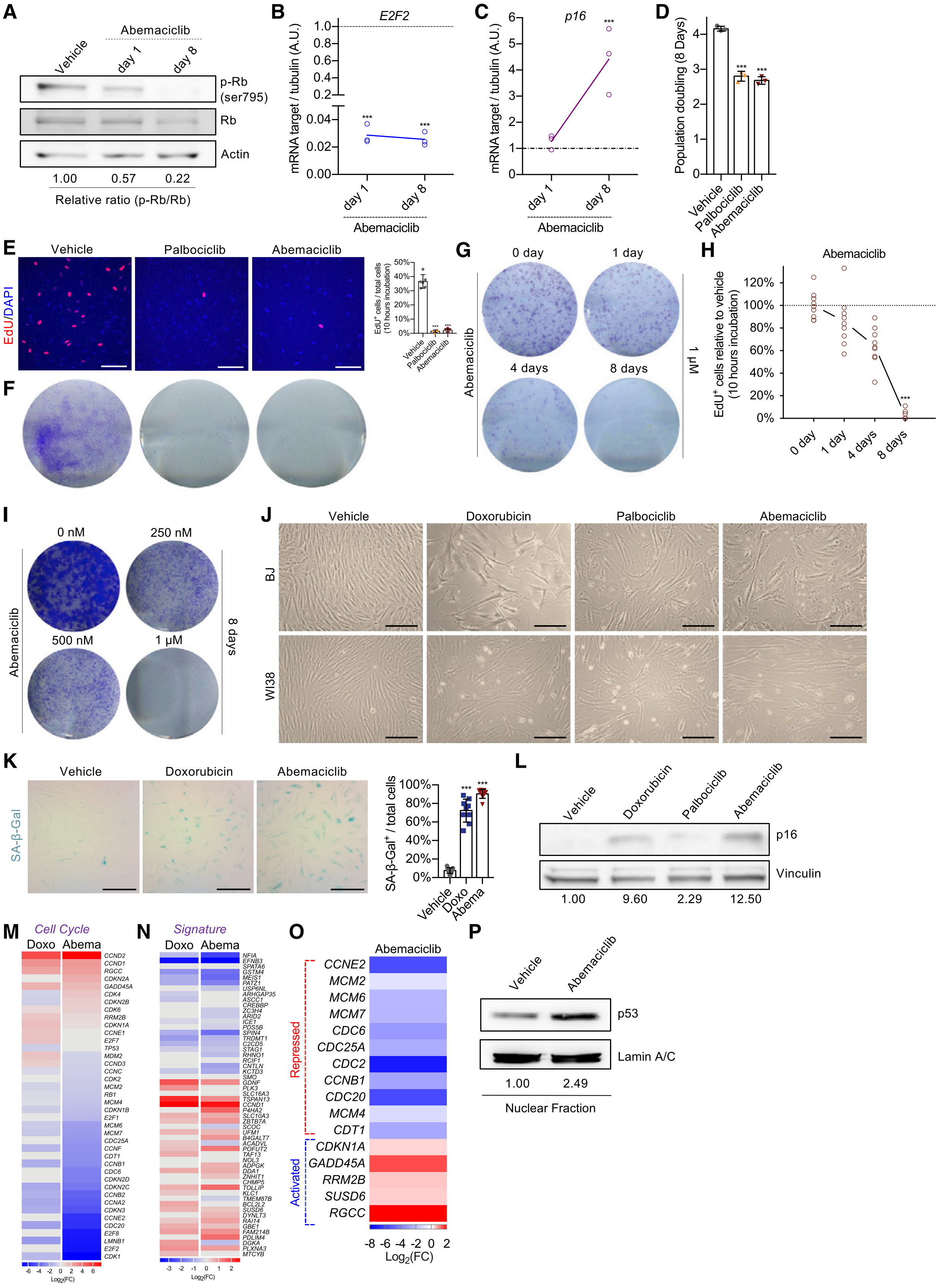

Figure EV1.

 © 2022 The Authors

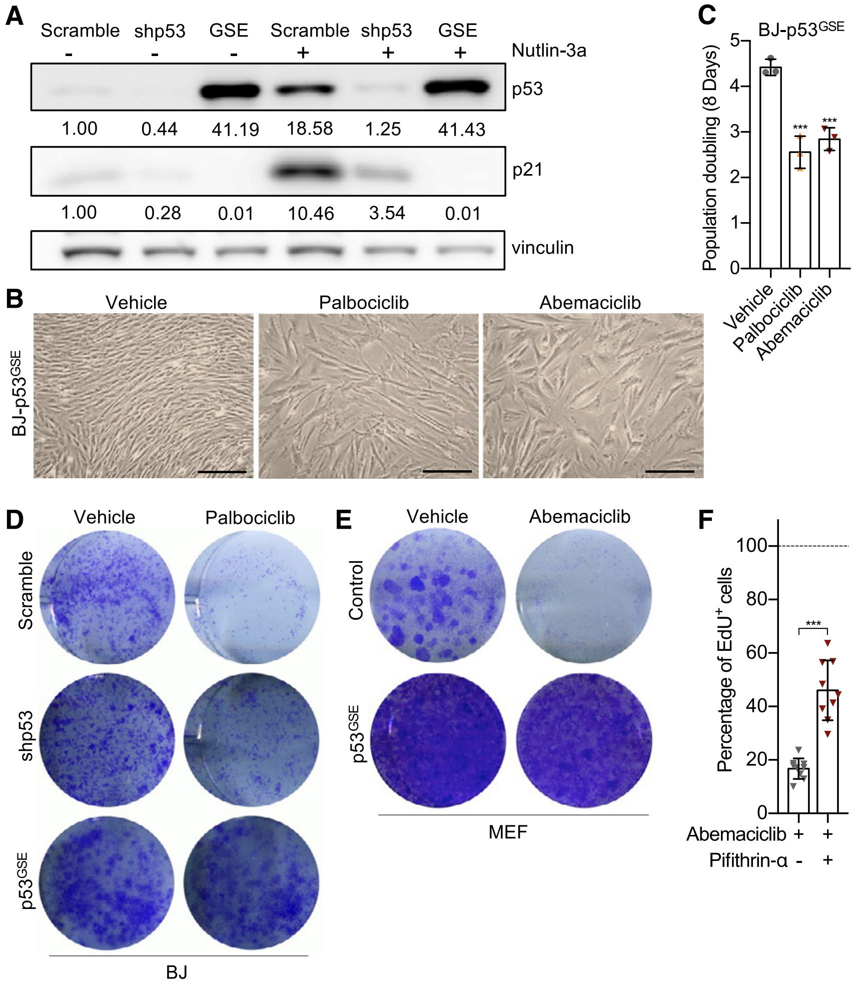

Figure EV2. CDK4/6i induces cellular senescence dependent on p53.

A Immunoblot of p53 on cells of indicated genotypes and treatments.

B Representative images of $p53^{GSE}$ BJ cells at the end of the 8-day indicated treatments (scale bar, 1 mm; $n = 3$ independent experiments).

C 8-day population doubling of treated $p53^{GSE}$ BJ cells ($n = 3$ independent experiments).

D 3×10^3 vehicle or palbociclib-treated (1 μM for 8 times in 24 h) scramble/shp53/ p53GSE BJ cells were replated in 6-well dishes, cultured for 8 days, and stained with 0.2% crystal violet ($n = 3$ independent experiments).

E 3×10^3 vehicle- or abemaciclib-treated (4 μM for 8 times in 24 h) WT/p53GSE MEFs were replated in 6-well dishes, cultured for 8 days and stained with 0.2% crystal violet ($n = 3$ independent experiments).

F BJ cells were treated with abemaciclib ± pifithrin-α and EdU staining performed at 8 dpt ($n = 9$ samples from 3 independent experiments).

Data information: Data are means ± SD. One-way ANOVA (C). Unpaired two-tailed *t*-test (F). $^{***}P < 0.001$.

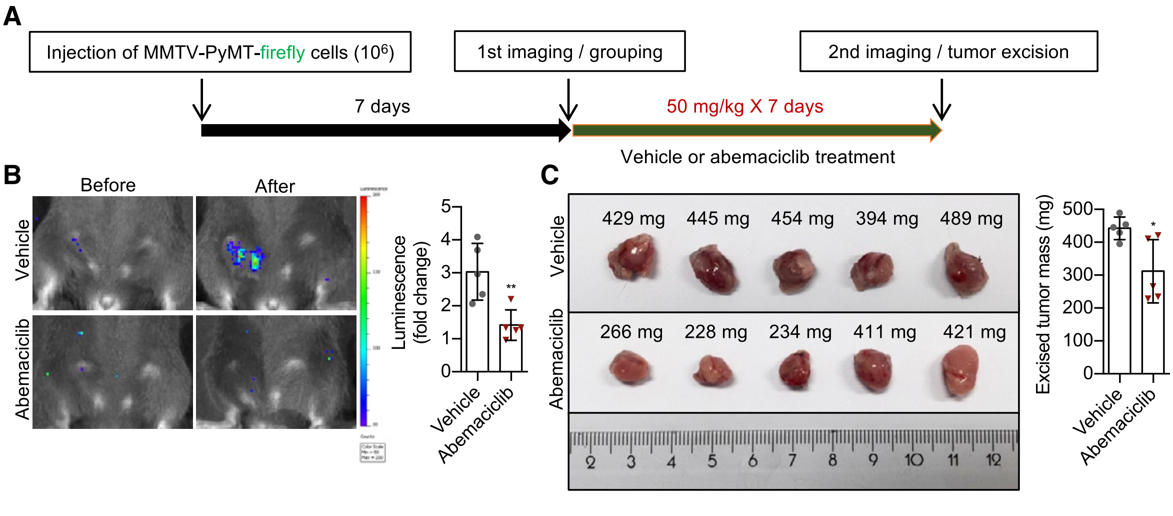

Figure EV3.

Figure EV3. Senescence-inducing dose of abemaciclib inhibits tumor growth.

A Scheme of abemaciclib treatments for MMTV-PyMT-firefly breast cancer mouse model *in vivo*.

B Female p16-3MR mice bearing MMTV-PyMT-firefly tumors in mammary fat pad were treated with vehicle (PBS, 7 consecutive days) or abemaciclib (50 mg/kg in PBS, 7 consecutive days). The mice were injected with D-Luciferin, and bioluminescence was visualized/quantified by the IVIS spectrum *in vivo* imaging system before and after abemaciclib treatments, as shown by representative images and quantification (n = 5 mice/group).

C Excised tumors and quantification of tumor weights (n = 5 mice/group).

Data information: Data are means ± SD. Unpaired two-tailed t-test (B and C). *$P < 0.05$ and **$P < 0.01$.

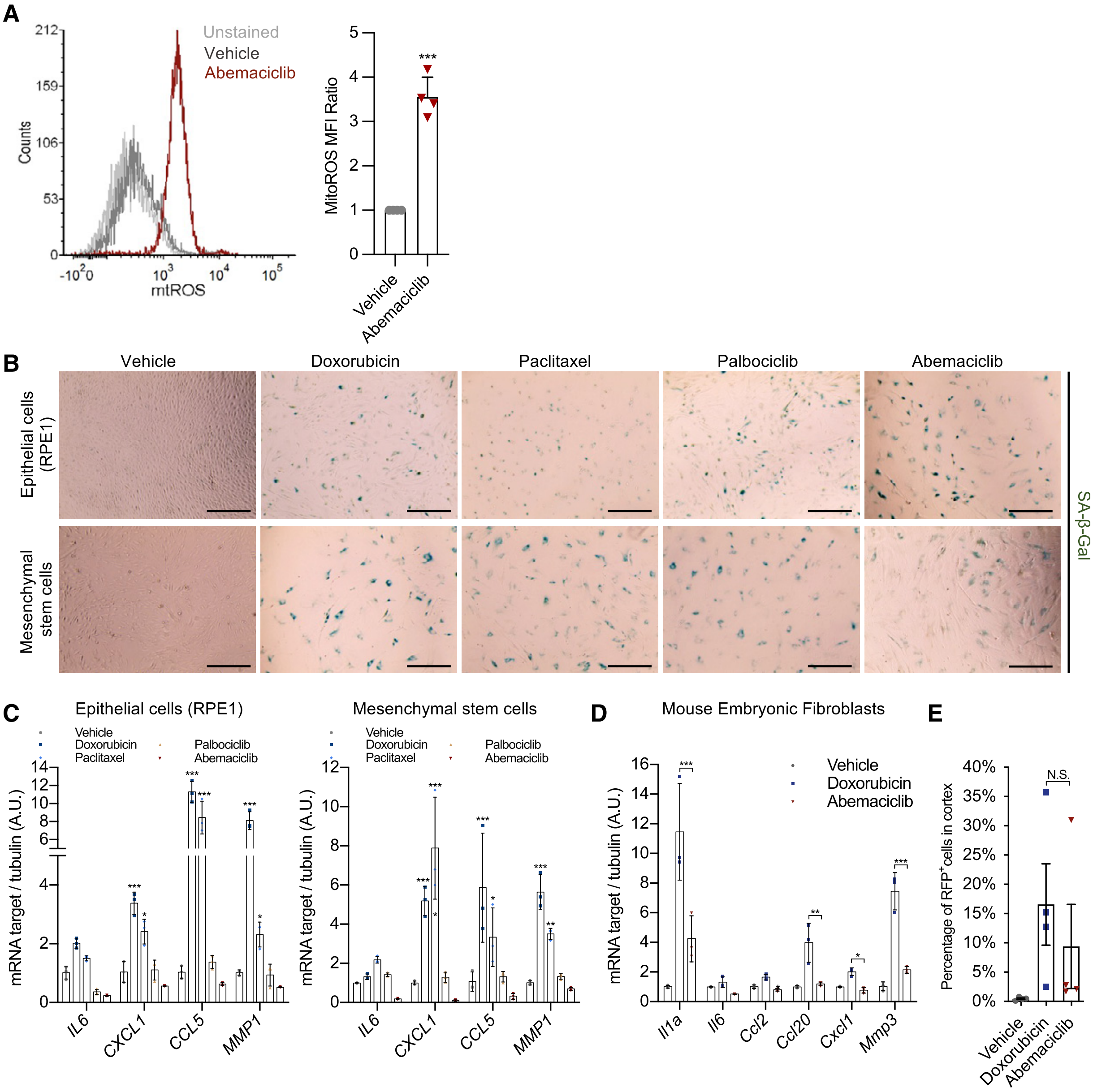

Figure EV4.

 © 2022 The Authors

Figure EV4. CDK4/6i induces cellular senescence without pro-inflammatory SASP.

A BJ cells were treated with vehicle or abemaciclib and stained for mitochondria ROS. Relative values of ROS level were plotted (n = 4 independent experiments).
B Human hTERT-RPE1 and lung mesenchymal stem cells were treated with vehicle (DMSO for 8 times in 24 h) or doxorubicin (250 nM for 24 h) or paclitaxel (50 nM for 24 h) or palbociclib (1 μM for 8 times in 24 h) or abemaciclib (1 μM for 8 times in 24 h). At 8 dpt, treated cells were fixed and stained for SA-β-gal (scale bar, 1 mm; n = 3 independent experiments).
C At 8 dpt, RNA was isolated from treated cells and indicated NF-κB-associated SASP (NASP) genes were quantified by qRT–PCR relative to tubulin (n = 3 independent experiments).
D qRT–PCR of indicated genes was performed using mouse embryonic fibroblast (MEFs) 8 days after the indicated treatments (n = 3 independent experiments).
E RFP^+ cells were sorted from renal cortex of doxorubicin- or abemaciclib-treated p16-3MR mice. The percentage of RFP^+ cells in total cells was plotted (n = 4 mice/group).

Data information: Data are means ± SD. Two-way ANOVA (C and D). One-way ANOVA (E). *$P < 0.05$, **$P < 0.01$, and ***$P < 0.001$, N.S. = not significant.

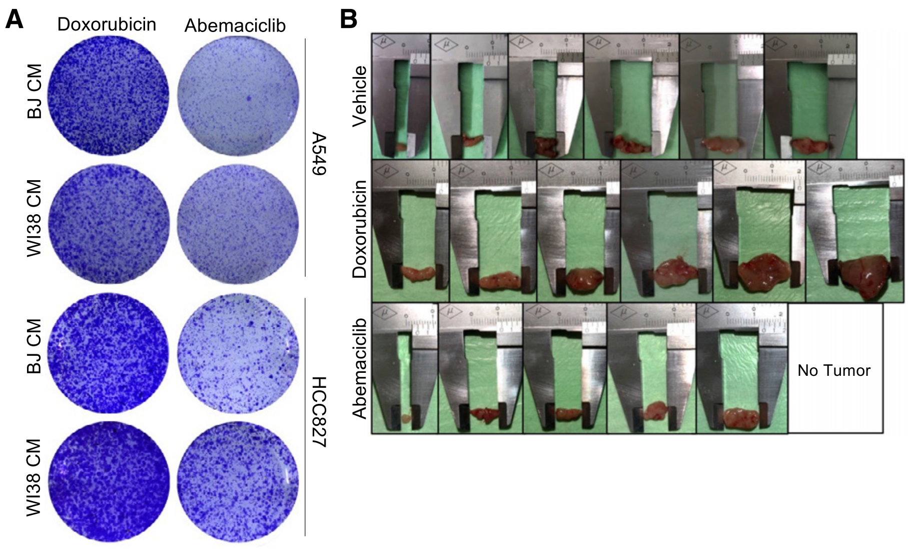

Figure EV5. p53-associated SASP lacks pro-tumorigenic properties.

A A549 and HCC827 lung cancer cells were incubated with drug-induced BJ or WI38 serum-free CM for 45 h. Cells were replated for colony formation assay for 8 days (n = 3 independent experiments).
B Excised tumors from Fig 5D (n = 6 mice/group).

© 2022 The Authors
