# Supplementary material for: Pharmacological CDK4/6 inhibition reveals a p53‐dependent senescent state with restricted toxicity
Source: EMBO J. 2022 Jan 5;41(6):e108946. doi: 10.15252/embj.2021108946 (PMC8922251; doi:10.15252/embj.2021108946)
Supplement: Supplementary file 5 [file EMBJ-41-e108946-s004.pdf]

THE EMBO JOURNAL

# Pharmacological CDK4/6 inhibition reveals a p53-dependent senescent state with restricted toxicity

Boshi Wang, Marta Varela-Eirin, Simone Brandenburg, alejandra HErnandez-Segura, Thijmen van Vliet, Elisabeth Jongbloed, Saskia Wilting, Naoko Ohtani, Agnes Jager, and Marco Demaria
**DOI: 10.15252/embj.2021108946**

*Corresponding author(s): Marco Demaria (m.demaria@umcg.nl)*



## Review Timeline:

| | |
|---|---|
| Submission Date: | 10th Jun 21 |
| Editorial Decision: | 5th Aug 21 |
| Revision Received: | 25th Oct 21 |
| Editorial Decision: | 26th Nov 21 |
| Revision Received: | 29th Nov 21 |
| Accepted: | 2nd Dec 21 |

*Editor: Hartmut Vodermaier*


## Transaction Report:

(Note: With the exception of the correction of typographical or spelling errors that could be a source of ambiguity, letters and reports are not edited. Depending on transfer agreements, referee reports obtained elsewhere may or may not be included in this compilation. Referee reports are anonymous unless the Referee chooses to sign their reports.)

Thank you again for your patience during our arbitrating review of your transferred manuscript on p53-dependent senescence programs upon CDK4/6 inhibition. I have now heard back from two experts, who looked into the study as well as into the transferred previous referee reports and your response to them. Given their overall interest and generally supportive comments, we would be happy to pursue this work further for EMBO Journal publication, pending revision along the lines suggested in your tentative response letter and also taking the additional thoughts of our arbitrating referees on board.

To recapitulate what the key points would be:

- testing a few additional NASP factors (orig. ref 1/pt 3)
- briefly test a Cdk4/6 inhibitor concentration curve (orig. ref 2/pt 1 and arbitrating ref 1)
- Orig Ref pt 2: add new data as proposed in your response, AND add some discussions & thoughts on how CDK4/6 inhibition might activate p53 in the absence of DNA damage or ROS (cf. arbitrating ref 2).
- including the proposed experiment to isolate induced senescent cells as proposed in response to orig. ref 2/pt 6 and further elaborated on by arbitrating ref 1.
- add some discussions and thoughts on what mechanisms may be involved in clearing CDK4/6i-induced senescent cells despite the lack of NASP (see arbitrating referee 2); might it be possible to get some deeper insight by adding additional mid-time points in the IHC clearance time course in current Fig. 5J?

Since it is our policy to consider only a single round of major revision, it will be important to comprehensively answer to all the points raised at the time of resubmission; I would be happy to discuss the time line for the revision work with me once you had the time to consider this letter. I can also remind of our 'scooping protection', which will allow you to finish dedicated revision experiments without the danger of losing novelty upon publication of related/competing work here or elsewhere.

Further information on preparing and uploading a revised manuscript can be found below and in our Guide to Authors. Thank you again for the opportunity to consider this work for The EMBO Journal, and I look forward to your revision.

REFEREE REPORTS

---------------------

Arbitrating Referee #1:

1. I think the study is interesting and, in general, appropriate for EMBO Journal.

2. Some of the points raised by Rev 2 are valid and should be addressed in the revision. In addition to what the authors suggested to do, which is mostly fine, I'd suggest a few simple experiments that would address the concern of this reviewer:

For point 1 - I suggest doing a concentration curve from 200 nM to 1uM and check which dose induces irreversible arrest following 8 days of treatment.

For point 6 - the authors already suggested that they will do the experiment. It is important to look at cells from different tissues in this experiment to see the real picture. This experiment will prove (or not) the relevance of the finding in vivo. I suggest authors focus on this and provide comprehensive information on this point.

For the other points raised by the reviewer, I suggest the authors downtone some of their conclusions instead of arguing with the reviewer.

Arbitrating Referee #2:

CDK4/6i (or p16)-induced senescence is a unique type, lacking a typical inflammatory sasp and persistent DNA damage response (two major effectors in senescence). In this study, Wang and colleagues identify and characterise p53-dependent sasp in such senescence in (non-cancer cells). this seems to be a subset of typical sasp. rather unexpectedly, these 'cold-senescence' still appears to get cleared in vivo.

The authors have addressed most of the reviewers' questions. one outstanding one is how CDK4/6i activates p53 without DDR. This is certainly an interesting but challenging question. The authors appear more concerned with the functional relevance, which is also very important. General and tumorigenic side effects of CDK4/6i-induced senescence appear to be lower than genotoxic chemotherapies, reinforcing the potential advantage of this therapeutic strategy in cancer. In this context, I would be very curious how CDK4/6i-senescent cells are cleared. Is this immune-mediated, or do they just die? This might open up new interesting questions for the future.

**To recapitulate what the key points would be:**

---

**- testing a few additional NASP factors (orig. ref 1/pt 3)**

***orig. ref 1/pt 3.*** *Although results in figure 4L are intriguing, it is unclear why only 2 NASP factors were studied (and why these 2): were also others tested, what was their variation?*

AUTHORS: We have now expanded the analysis of NASP factors and performed ELISA for CCL2 and MMP1. As expected from our hypothesis and as observed for CXCL1 and CCL5, only paclitaxel-treated patients showed elevated levels post-treatment. These new data were incorporated in Fig 3L.

** Figures for referees not shown.

---

**- briefly test a Cdk4/6 inhibitor concentration curve (orig. ref 2/pt 1 and arbitrating ref 1)**

***orig. ref 2/pt 1.*** *cdk4/6 inhibitor drugs, while commonly used at 1 uM, are active at lower concentrations (100nM-200nM maximum) even in tissue culture. These nM doses are what occurs at physiologic levels in human beings and mice.*

AUTHORS: We agree that certain cancer cell lines seem to respond to lower concentrations of CDK4/6 inhibitors, but also that in many studies a significant cytostatic effect is reached only at high concentrations (2-5 uM). It is important to note that the mean concentration of the active metabolites of abemaciclib achieved in patient plasma is approximately 1 uM (Ref: https://www.cell.com/cancer-cell/fulltext/S1535-6108(17)30509-3). To add to the selection of 1 uM as our working concentration, we have studied a titration of abemaciclib on irreversible growth arrest. We treated BJ cells with 250nM, 500nM or 1uM abemaciclib for 8 times 24 hours, and then replated the cells for colony formation assays. As shown below, partial effects on proliferation were observed at 250 and 500 nM, while strong effects were achieved at 1uM. These data are now included in Fig EV1I.

** Figures for referees not shown.

---

**- Orig Ref pt 2: add new data as proposed in your response, AND add some discussions & thoughts on how CDK4/6 inhibition might activate p53 in the absence of DNA damage or ROS (cf. arbitrating ref 2).**

***orig ref 2/pt 2**. How do you get a p53 response? Does the drug induce ROS? Does it cause DNA damage? If it causes DNA damage, then based on your citations of the literature, why would it not have an NFkB response? It is insufficient in a molecularly, mechanism-oriented, manuscript to report it without providing an understanding of it. Perhaps you change the nature of the p53 response, but again the data is somewhat deficient for this. One example, is in your figure 1 you score p53 binding to target loci, but you do not compare this binding to that of a clearly dependent DNA damage p53 response here, but when reading other outputs, such as transcription in figure 3 you do that control. Such "cherry-picking" of data does not make for a persuasive argument that p53 is even involved.*

AUTHORS: We have now performed analysis of mitochondrial ROS, and observed upregulation in cells treated with abemaciclib (Fig EV4A). However, this is not sufficient to induce a significant DDR, thus the absence of NFkB signaling. These data were already shown in the original submission as Fig 3A-B. In addition to the ROS data, we have also measured the level of nuclear p53, showing an increase in cells treated with abemaciclib (Figure EV1P).

In order to compare p53 binding to target loci in DNA damage models, we have performed a ChIP experiment including doxorubicin-treated cells. As we show in Figure, the increase in p53-binding activity is similar between abemaciclib and doxorubicin treatment.

** Figures for referees not shown.

**- including the proposed experiment to isolate induced senescent cells as proposed in response to orig. ref 2/pt 6 and further elaborated on by arbitrating ref 1.**

***orig. ref 2/pt 6***. *Rather than look indirectly for cytokines in serum, why don't you treat mice with cdk4/6 inhibitors and look at the stromal cells for evidence of this event, perhaps the proliferating epithelial cells in the gut, or the mesenchymal cells during wound repair. Maybe you could isolate them and use single cell seq to define the phenotype and its relation to a p53-dependent non-inflammatory phenotype in cultured cells. Alternatively, you might come up with a way to show that a cell has "stable arrest induced by cdk4/6 inhibitor" by creative use of fluorescent indicators that monitor the expression programs and DNA replication after drug withdrawal.*

AUTHORS: In order to address SASP expression directly in vivo, we treated the p16-3MR mice with vehicle, doxorubicin or abemaciclib and sorted the RFP+ (p16+) cells from the kidney cortex. RNA was isolated and qPCR targeting pro-inflammatory SASP was performed. Data indicate a high expression of pro-inflammatory SASP factors in cells isolated from doxorubicin-treated but not from abemaciclib-treated mice. These data re now in Fig 3K and EV4E.

** Figures for ~~re~~ferees not shown.

**- add some discussions and thoughts on what mechanisms may be involved in clearing CDK4/6i-induced senescent cells despite the lack of NASP (see arbitrating referee 2); might it be possible to get some deeper insight by adding additional mid-time points in the clearance time course in current Fig. 5J?**

AUTHORS: We have now added a mid-time point (7 dpt) in the time course of the clearance experiment, and added these data in the quantification graph of Fig 5J.

** Figures for referees not shown.

Thank you again for submitting your revised manuscript for our consideration, and apologies for the delayed re-review, during which both arbitrating referees have now assessed the data added subsequent to your earlier tentative response to the previous reviews, as well as your answers to the points they had emphasized. As you will see from the comments copied below, arbitrator 1 was not satisfied with all revisions, necessitating careful further consultations both within our team and with arbitrator 2.

The first issue raised by arbitrator 1 concerns the inhibitor concentration, which the reviewer considers too high to be clinically significant. Arbitrator 2 has now taken a detailed second look at this (see additional comments below), and while appreciating the reason for the concern, feels that the new data would still support senescence induction in a physiological range.

The second criticism of arbitrator 1 concerns the new senescent cell isolation experiments (Fig 3K and EV4E), as they have only been done from kidney but not other tissues. I appreciate that the initial revision proposal had not clearly specified which tissues exactly you were planning to analyze, and the referees had not explicitly excluded kidney as a relevant tissue to use either. Nevertheless, given that also arbitrator 2 agrees that the study would be strengthened by inclusion of data from additional tissues, I would strongly encourage you to add any such data that you may already have.

Finally, I appreciate your evidence for PASP being directly due to p53 transcriptional activity, and p53 activation not being due to DNA damage or ROS. But I still miss any thoughts on what else might then be mediating p53 activation upstream of the PASP? I.e., an (even if speculative) answer to original referee 2's question "How do you get a p53 response?"
=> even if this may already be the topic of follow-up work, please do add some concrete thoughts on how CDK4/6 inhibition might cause p53 activation (as asked in my previous decision letter) to the discussion.

In conclusion, we decided that pending adequate re-revision, we would consider the study further for eventual publication in The EMBO Journal. In addition to paying attention to the above points, this final version should also incorporate the following editorial points:

------------------------------------------------

Arbitrating Referee #1:

I acknowledge that the authors perform some of the suggested experiments in regard to points 1 and 6 of rev2 that I asked to address in my previous review. Below is the analysis of the results I see.
Regarding point 1:
A concern is that the concentration used (1 uM) is way above what happens in patients and therefore observed results might not be relevant to the patients. The authors perform the experiment with lower concentrations of the drug and the results show that these concentrations do not induce senescence as the arrest is reversible. The authors originally cited a paper in Cancer Cell and suggested that this paper shows that 1uM is the concentration in patients. I've looked at this paper and surprisingly found that there were no measurements of plasma concentration of the drug in patients in this paper. The citation of this paper was, apparently, misleading.
The studies with patients (https://cancerdiscovery.aacrjournals.org/content/6/7/740 ;

https://clincancerres.aacrjournals.org/content/26/20/5310 ) clearly show that the concentration in the plasma and other internal body fluids is indeed 100nm and can reach up to 500nM only at the maximum tolerated dose, which is rarely used.
The new results presented by the authors show that even at 500nM the cells resume proliferation after removal of the drug - means they are not senescent and thus strikingly different from the cells treated with 1uM.
Unfortunately, all the above shows that the concern of the reviewer was valid and the concentration used in the study is not clinically relevant. Therefore, all the conclusions regarding relevance to the patients do not stand.

Regarding point 6:
The question was if there is an increase in senescent cells with the described properties in different tissues following the drug treatment. The authors continue to focus on one tissue - the kidney. The kidney is an important metabolic organ and is responsible for the removal of all the metabolites of the drug and partially the drug itself. Therefore, concentrating at the kidney provides only a limited picture. The authors themselves suggested that they will isolate cells from different tissues. Apparently, the result of such experiments is not shown and thus raises the concern of the appearance of the relevant cells in tissues even higher.

Arbitrating Referee #2:

The authors have added new data addressing the remaining issues.

ADDITIONAL CROSS-COMMENTS on Arbitrator 1:

The first point seems to come down to the question about 'active metabolites of abemaciclib' vs 'parent abemaciclib'.

Gong et al (the paper cited by the authors) describe that Abemaciclib mean steady-state plasma concentrations range from 0.4 to 0.6 μM. This is based on Patnaik et al., 2016 (cited by reviewer 1). I have to say it was not easy to find the exact numbers in this paper (at least to me). But let's say it is correct, this seems to represent the 'abemaciclib' parent drug, and Gong et al doubled the concentration to reflect the 'abemaciclib' parent drug + active metabolites, leading to 1uM as an 'upper threshold' for their in vitro (cell lines) screens. Thus, based on this argument, 1uM in vitro is high but may not be too far from the physiological level. However, it is true that the other paper, which was cited by reviewer 1, estimates plasma concentration of 'active metabolites' a bit lower. It is hard to directly compare between total active metabolites/parent drug in the plasma and parent drug in culture media.

Having said this, their new colony formation assay data using lower doses show substantial differences, thus lower doses do induce senescence (although not 100%). Additionally, the authors might argue that they do find senescence in mice using the tolerable concentration (50mg/kg).

I find the second point (they only used kidneys) is more problematic. Sorry, I didn't pick this up. But I would think they must have data from other issues already. It should be easy for them to add those data.

The first issue raised by arbitrator 1 concerns the inhibitor concentration, which the reviewer considers too high to be clinically significant. Arbitrator 2 has now taken a detailed second look at this (see additional comments below), and while appreciating the reason for the concern, feels that the new data would still support senescence induction in a physiological range.

AUTHORS: we agree that the debate about abemaciclib concentration remains open and important. However, as we have mentioned in the manuscript, we have tried to mimic a clinically-relevant situation, even if the concentration used for each dosage is in the high-end of the spectrum. On this point, we would also like to add that human patients are treated for much longer periods of time than 7 days (in the clinical trial MonarchE patients were treated up to 2 years – see https://ascopubs.org/doi/10.1200/JCO.20.02514).

The second criticism of arbitrator 1 concerns the new senescent cell isolation experiments (Fig 3K and EV4E), as they have only been done from kidney but not other tissues. I appreciate that the initial revision proposal had not clearly specified which tissues exactly you were planning to analyze, and the referees had not explicitly excluded kidney as a relevant tissue to use either. Nevertheless, given that also arbitrator 2 agrees that the study would be strengthened by inclusion of data from additional tissues, I would strongly encourage you to add any such data that you may already have.

AUTHORS: during the first round of revision we did not indeed mention any particular tissue. The sorting of RFP+ (p16+) cells from tissues is technically challenging and we can process only one tissue/mouse. The decision to sort from kidneys is due to our previous studies (Demaria M et al, Cancer Discovery, 2017; Van Vliet et al, Mol Cell, 2021) which indicated the kidney being a tissue accumulating premature senescence and SASP in response to exposure to genotoxic chemotherapy. Analysis of another tissue would require a new cohort of mice requiring additional resources and raising several ethical concerns. Thus, it is not possible for us at this stage to add more data to this point.

Finally, I appreciate your evidence for PASP being directly due to p53 transcriptional activity, and p53 activation not being due to DNA damage or ROS. But I still miss any thoughts on what else might then be mediating p53 activation upstream of the PASP? I.e., an (even if speculative) answer to original referee 2's question "How do you get a p53 response?"
=> even if this may already be the topic of follow-up work, please do add some concrete thoughts on how CDK4/6 inhibition might cause p53 activation (as asked in my previous decision letter) to the discussion.

AUTHORS: we have now added this point to the discussion part. In particular, we are suggesting that future studies should aim at understanding how p53 is activated, but also at the role of epigenetics and accessibility to p53 target genes.

**ACCEPTED** **2nd Dec 2021**

Thank you for submitting your final revised manuscript for our consideration. I am pleased to inform you that we have now accepted it for publication in The EMBO Journal.

EMBO PRESS

YOU MUST COMPLETE ALL CELLS WITH A PINK BACKGROUND ↓

PLEASE NOTE THAT THIS CHECKLIST WILL BE PUBLISHED ALONGSIDE YOUR PAPER

Corresponding Author Name: Marco Demaria
Journal Submitted to: EMBO Journal
Manuscript Number: EMBOJ-2021-108946

## USEFUL LINKS FOR COMPLETING THIS FORM

http://www.antibodypedia.com
http://1degreebio.org
http://www.equator-network.org/reporting-guidelines/improving-bioscience-research-report
http://grants.nih.gov/grants/olaw/olaw.htm
http://www.mrc.ac.uk/Ourresearch/Ethicsresearchguidance/Useofanimals/index.htm
http://ClinicalTrials.gov
http://www.consort-statement.org
http://www.consort-statement.org/checklists/view/32-consort/66-title
http://www.equator-network.org/reporting-guidelines/reporting-recommendations-for-tumo
http://datadryad.org
http://figshare.com
http://www.ncbi.nlm.nih.gov/gap
http://www.ebi.ac.uk/ega
http://biomodels.net/
http://biomodels.net/miriam/
http://jjj.biochem.sun.ac.za
https://osp.od.nih.gov/biosafety-biosecurity-and-emerging-biotechnology/
http://www.selectagents.gov/

## Reporting Checklist For Life Sciences Articles (Rev. June 2017)

This checklist is used to ensure good reporting standards and to improve the reproducibility of published results. These guidelines are consistent with the Principles and Guidelines for Reporting Preclinical Research issued by the NIH in 2014. Please follow the journal's authorship guidelines in preparing your manuscript.

### A- Figures

### 1. Data

### The data shown in figures should satisfy the following conditions:

- the data were obtained and processed according to the field's best practice and are presented to reflect the results of the experiments in an accurate and unbiased manner.
- figure panels include only data points, measurements or observations that can be compared to each other in a scientifically meaningful way.
- graphs include clearly labeled error bars for independent experiments and sample sizes. Unless justified, error bars should not be shown for technical replicates.
- if n< 5, the individual data points from each experiment should be plotted and any statistical test employed should be justified
- Source Data should be included to report the data underlying graphs. Please follow the guidelines set out in the author ship guidelines on Data Presentation.

### 2. Captions

### Each figure caption should contain the following information, for each panel where they are relevant:

- a specification of the experimental system investigated (eg cell line, species name).
- the assay(s) and method(s) used to carry out the reported observations and measurements
- an explicit mention of the biological and chemical entity(ies) that are being measured.
- an explicit mention of the biological and chemical entity(ies) that are altered/varied/perturbed in a controlled manner.
- the exact sample size (n) for each experimental group/condition, given as a number, not a range;
- a description of the sample collection allowing the reader to understand whether the samples represent technical or biological replicates (including how many animals, litters, cultures, etc.).
- a statement of how many times the experiment shown was independently replicated in the laboratory.
- definitions of statistical methods and measures:
  - common tests, such as t-test (please specify whether paired vs. unpaired), simple χ2 tests, Wilcoxon and Mann-Whitney tests, can be unambiguously identified by name only, but more complex techniques should be described in the methods section;
  - are tests one-sided or two-sided?
  - are there adjustments for multiple comparisons?
  - exact statistical test results, e.g., P values = x but not P values < x;
  - definition of 'center values' as median or average;
  - definition of error bars as s.d. or s.e.m.

Any descriptions too long for the figure legend should be included in the methods section and/or with the source data.

**In the pink boxes below, please ensure that the answers to the following questions are reported in the manuscript itself. Every question should be answered. If the question is not relevant to your research, please write NA (non applicable). We encourage you to include a specific subsection in the methods section for statistics, reagents, animal models and human subjects.**

### B- Statistics and general methods

Please fill out these boxes ↓ (Do not worry if you cannot see all your text once you press return)

| | |
|---|---|
| 1.a. How was the sample size chosen to ensure adequate power to detect a pre-specified effect size? | The sample size was determined based on experience of former studies for animal experiments. For in vitro experiments, at least 3 independent experiments were performed for each result. |
| 1.b. For animal studies, include a statement about sample size estimate even if no statistical methods were used. | We estimate the sample size for the animal studies based on former studies and publications. |
| 2. Describe inclusion/exclusion criteria if samples or animals were excluded from the analysis. Were the criteria pre-established? | Exclusion criteria was pre-established for the animals studies (i.e. sick animals or animals with significant weight difference will be excluded). But in this study, no animal was excluded when the experiments were done. |
| 3. Were any steps taken to minimize the effects of subjective bias when allocating animals/samples to treatment (e.g. randomization procedure)? If yes, please describe. | Randomization was applied in most of the animal experiments (drug treatments for healthspan and SASP analysis). For tumor-bearing experiments, the mice were divided into different groups based on the tumor sizes to make a even distribution, before the treatments started. |
| For animal studies, include a statement about randomization even if no randomization was used. | Randomization was used in this study. |
| 4.a. Were any steps taken to minimize the effects of subjective bias during group allocation or/and when assessing results (e.g. blinding of the investigator)? If yes please describe. | The researchers were blinded for tumor measurements and healthspan tests in the animal experiments. For in vitro experiments, blinding cannot be achieved in any single experiment since usually only one researcher is involved. But independent experiments were performed by different resarchers to repeat the findings. |
| 4.b. For animal studies, include a statement about blinding even if no blinding was done | The researchers were blinded for tumor measurements and healthspan tests in the animal experiments. |
| 5. For every figure, are statistical tests justified as appropriate? | Yes, statistical tests were listed in the figure legends |
| Do the data meet the assumptions of the tests (e.g., normal distribution)? Describe any methods used to assess it. | Yes. Graphpad prism was used to determine the tests. |
| Is there an estimate of variation within each group of data? | Yes |
| Is the variance similar between the groups that are being statistically compared? | Yes |

### C- Reagents

| | |
|---|---|
| 6. To show that antibodies were profiled for use in the system under study (assay and species), provide a citation, catalog number and/or clone number, supplementary information or reference to an antibody validation profile. e.g., Antibodypedia (see link list at top right), 1DegreeBio (see link list at top right). | Immunodetection was performed by standard procedures of p16/Ink4a (clone EPR1473, cat# ab108349, Abcam), p21 (clone C-19, cat# sc-397, Santa Cruz Biotechnology), p53 (clone DO-1, cat# sc-126, Santa Cruz Biotechnology), Rb (clone 4H1, cat# 9309, Cell Signaling Technology) and phospho-Rb (ser795, cat# 9301, Cell Signaling Technology). The antibodies for the proteins of interest were used in 1:1000 dilution in 5%milk/TBST (Tris-buffered Saline with 0.1% Tween20) and incubated overnight at 4 degrees Celsius. Immunodetection of vinculin (cat# V9131, Sigma-Aldrich) or β-Actin (clone C4, cat# 08691001, MP Biomedicals) or Lamin A/C (Santa Cruz, sc-71481) was performed as loading control. |
| 7. Identify the source of cell lines and report if they were recently authenticated (e.g., by STR profiling) and tested for mycoplasma contamination. | BJ (CRL-2522), WI38 (CRL-7728), MMTV-PyMT (CRL-3278), A549 (CCL-185), HCC827 (CRL-2868), MCF7 (HTB-22) and hTERT-RPE1 (CRL-4000) cells were purchased from ATCC. Primary human MSCs were a gift from Prof. Irene Heijink (University Medical Center Groningen, The Netherlands). MEFs were generated from 13.5-day wild-type embryos. MDFs were isolated from the dorsal skin of 3-month-old p53-null mice or wild-type littermates and a gift from Prof. Paul Hasty (University of Texas Health Science Center at San Antonio, USA). Cells were not re-authenticated by the laboratory but were regularly monitored for mycoplasma contaminations (once per month). No cell line used was listed in the database of commonly misidentified cell lines maintained by ICLAC. All cells were cultured in DMEM-GlutaMAX (Thermo Fisher) medium supplemented with 10% fetal bovine serum (GE Healthcare Life Sciences) and 1% penicillin-streptomycin (Lonza). All the human and mouse normal primary cells were maintained in 5% O2, 5% CO2, 37 ºC incubators and all cancer cells were maintained in 20% O2, 5% CO2, 37 ºC incubators. |

* for all hyperlinks, please see the table at the top right of the document

## D- Animal Models

| | |
|---|---|
| 8. Report species, strain, gender, age of animals and genetic modification status where applicable. Please detail housing and husbandry conditions and the source of animals. | All the mice were maintained in the central animal facility (CDP) of University Medical Center Groningen (UMCG) under standard conditions. Animals are group housed in the conventional housing for WT mice and p16-3MR mice or IVC unit for the nude mice. Both male and female WT C57/bl6 mice and p16-3MR mice are used in this study. WT C57/bl6 mice and nude mice were purchased from Charles River laboratory. p16-3MR mice were bred in-house. |
| 9. For experiments involving live vertebrates, include a statement of compliance with ethical regulations and identify the committee(s) approving the experiments. | All the experiments were approved by the Central Authority for Scientific Procedures on Animals in the Netherlands. |
| 10. We recommend consulting the ARRIVE guidelines (see link list at top right) (PLoS Biol. 8(6), e1000412, 2010) to ensure that other relevant aspects of animal studies are adequately reported. See author guidelines, under 'Reporting Guidelines'. See also: NIH (see link list at top right) and MRC (see link list at top right) recommendations. Please confirm compliance. | We followed the ARRIVE guidelines as much as possible. |

## E- Human Subjects

| | |
|---|---|
| 11. Identify the committee(s) approving the study protocol. | ErasmusMC |
| 12. Include a statement confirming that informed consent was obtained from all subjects and that the experiments conformed to the principles set out in the WMA Declaration of Helsinki and the Department of Health and Human Services Belmont Report. | Written informed consent was obtained from all patients. |
| 13. For publication of patient photos, include a statement confirming that consent to publish was obtained. | NA |
| 14. Report any restrictions on the availability (and/or on the use) of human data or samples. | N/A |
| 15. Report the clinical trial registration number (at ClinicalTrials.gov or equivalent), where applicable. | N/A |
| 16. For phase II and III randomized controlled trials, please refer to the CONSORT flow diagram (see link list at top right) and submit the CONSORT checklist (see link list at top right) with your submission. See author guidelines, under 'Reporting Guidelines'. Please confirm you have submitted this list. | N/A |
| 17. For tumor marker prognostic studies, we recommend that you follow the REMARK reporting guidelines (see link list at top right). See author guidelines, under 'Reporting Guidelines'. Please confirm you have followed these guidelines. | N/A |

## F- Data Accessibility

| | |
|---|---|
| 18: Provide a "Data Availability" section at the end of the Materials & Methods, listing the accession codes for data generated in this study and deposited in a public database (e.g. RNA-Seq data: Gene Expression Omnibus GSE39462, Proteomics data: PRIDE PXD000208 etc.) Please refer to our author guidelines for 'Data Deposition'.<br><br>Data deposition in a public repository is mandatory for:<br>a. Protein, DNA and RNA sequences<br>b. Macromolecular structures<br>c. Crystallographic data for small molecules<br>d. Functional genomics data<br>e. Proteomics and molecular interactions | Sequencing data is available on ArrayExpress under accession no. E-MTAB-7642. |
| 19. Deposition is strongly recommended for any datasets that are central and integral to the study; please consider the journal's data policy. If no structured public repository exists for a given data type, we encourage the provision of datasets in the manuscript as a Supplementary Document (see author guidelines under 'Expanded View' or in unstructured repositories such as Dryad (see link list at top right) or Figshare (see link list at top right). | NA |
| 20. Access to human clinical and genomic datasets should be provided with as few restrictions as possible while respecting ethical obligations to the patients and relevant medical and legal issues. If practically possible and compatible with the individual consent agreement used in the study, such data should be deposited in one of the major public access-controlled repositories such as dbGAP (see link list at top right) or EGA (see link list at top right). | Written informed consent was obtained from all patients in EMC. |
| 21. Computational models that are central and integral to a study should be shared without restrictions and provided in a machine-readable form. The relevant accession numbers or links should be provided. When possible, standardized format (SBML, CellML) should be used instead of scripts (e.g. MATLAB). Authors are strongly encouraged to follow the MIRIAM guidelines (see link list at top right) and deposit their model in a public database such as Biomodels (see link list at top right) or JWS Online (see link list at top right). If computer source code is provided with the paper, it should be deposited in a public repository or included in supplementary information. | NA |

## G- Dual use research of concern

| | |
|---|---|
| 22. Could your study fall under dual use research restrictions? Please check biosecurity documents (see link list at top right) and list of select agents and toxins (APHIS/CDC) (see link list at top right). According to our biosecurity guidelines, provide a statement only if it could. | NA |
